# Polyphenol-Gated Composite Electrolytes with Enhanced Cross-Phase Lithium-Ion Transport for Solid-State Lithium Batteries

**DOI:** 10.1007/s40820-026-02127-6

**Published:** 2026-03-10

**Authors:** Xiaoxiao Li, Minqiang Jiang, Kai Chen, Zhixiang Cai, Yingxin Zhang, Jiamei Luo, Lei Hou, Yazhou Zhou, Chao Zhang, Hui Zhang, Feili Lai, Yue-E Miao, Tianxi Liu, Klaus Müllen

**Affiliations:** 1 State Key Laboratory of Advanced Fiber Materials, College of Materials Science and Engineering, Donghua University, 2999 North Renmin Road, Shanghai, 201620 People’s Republic of China; 2 State Key Laboratory of Advanced Fiber Materials, College of Chemistry and Chemical Engineering, Donghua University, 2999 North Renmin Road, Shanghai, 201620 People’s Republic of China; 3 Nanotechnology Centre, Centre for Energy and Environmental Technologies, VŠB-Technical University of Ostrava, 17. Listopadu 2172/15, 70800 Ostrava-Poruba, Czech Republic; 4 Department of Chemistry, KU Leuven, Celestijnenlaan 200F, 3001 Louvain, Belgium; 5 State Key Laboratory of Metal Matrix Composites, School of Materials Science and Engineering, Shanghai Jiao Tong University, Shanghai, 200240 People’s Republic of China; 6 Key Laboratory of Synthetic and Biological Colloids, Ministry of Education, School of Chemical and Material Engineering, Jiangnan University, Wuxi, 214122 People’s Republic of China; 7 Department of Molecular Spectroscopy, Max Planck Institute for Polymer Research, Ackermannweg 10, 55128 Mainz, Germany

**Keywords:** Polyphenol-Gated Interface, Composite Solid-State electrolytes, Li^+^- Selective Transport, Polymer-Ceramic Interface, Solid-State Lihium-Matel Batteries

## Abstract

**Highlights:**

- A biomimetic polyphenol-gated strategy is proposed to promote interfacial Li^+^ - selective transport in composite solid electrolytes by chemically bonding the polymer matrix and ceramic nanofibers.
- The polyphenol interlayers serve as the chemical gates with –OH and –NH groups to immobilize lithium salt anions and carbonyl groups to coordinate Li^+^, thus lowering the energy barrier and promoting rapid Li^+^ transport at interface.
- The assembled Li||LiFePO_4_ batteries exhibits an impressive capacity of 151.6 mAh g^−1^ and long lifespan over 600 cycles.

**Abstract:**

Solid-state lithium (Li) batteries offer high-energy density and operational safety but face sluggish Li^+^ transport in polymer/ceramic composite solid-state electrolytes. Herein, we propose a bioinspired polyphenol-gated interfacial engineering that mimics ion-selective protein channels to enhance Li^+^-selective transport across the polymer–ceramic interface. Polyphenols such as polydopamine, poly-tannic acid, and poly-gallic acid chemically couple La_0.56_Li_0.33_TiO_3_ ceramic nanofibers and glycidyl polyether matrix. Within this interface, carbonyl groups selectively coordinate Li⁺ and facilitate directional migration. On the other hand, hydroxyl and amino groups immobilize anions via hydrogen bonding. This chemical gating nearly doubles interfacial Li^+^ concentration and boosts transference number to 0.68. The corresponding Li||LiFePO_4_ battery exhibits stable cycling over 600 cycles with 85.5% capacity retention at 1 C, while the pouch cell delivers reliable operation under mechanical stress caused by bending and puncturing. This work demonstrates that polyphenol-gated interfaces are essential for promoting selective and efficient cross-phase Li⁺ transport for high-performance solid-state lithium-metal batteries.

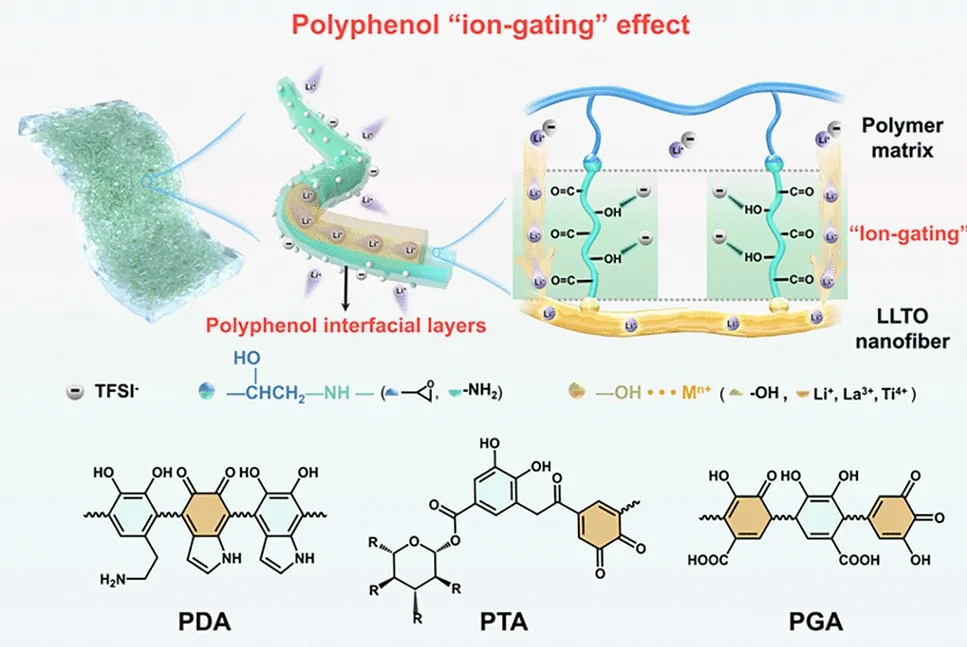

**Supplementary Information:**

The online version contains supplementary material available at 10.1007/s40820-026-02127-6.

## Introduction

All-solid-state lithium-metal batteries (ASSLMBs) have emerged as next-generation energy storage technology that combines high-energy density and safety. Unlike traditional LMBs that use flammable liquid electrolytes, ASSLMBs employ solid-state electrolytes that suppress leakage, reduce thermal runaway risks, and enable stable operation of Li anodes [1–3]. As a critical component, the solid electrolyte significantly influences overall battery performance. Polymer/ceramic composite solid-state electrolytes (CSEs) represent a leading strategy in this field. These systems integrate the flexibility and processability of polymers with high ion conductivity of ceramic fillers. Common polymers include poly(ethylene oxide) (PEO), poly(vinylidene fluoride) (PVDF), polyacrylonitrile (PAN), while typical ceramic fillers are garnet-type Li_7_La_3_Zr_2_O_12_ (LLZO), Li_6.75_La_3_Zr_1.75_Ta_0.25_O_12_ (LLZTO), and perovskite-type La_0.56_Li_0.33_TiO_3_ (LLTO) [4]. However, the electrochemical performance of these CSEs is still limited by inefficient Li⁺ transport across the polymer-ceramic interface.

To improve Li⁺ transport in CSEs, various concepts have been developed which can be categorized primarily into two approaches: (1) Enhancing the distribution of ceramic fillers in the polymer matrix. Uniform dispersion of fillers is critical for establishing continuous Li⁺-conducting networks and increasing the effective interfacial area for ion exchange. However, ceramic fillers often agglomerate. To address this issue, one method utilizes three-dimensional (3D) ceramic nanofiber frameworks (e.g., LLZO and LLTO) [5–8]. These frameworks provide interconnected ion pathways. However, the weak van der Waals forces at the polymer-ceramic interface limit ion exchange [9]. This reduces the local Li⁺ concentration at the ceramic surface and undermines the contribution of the ceramic phase to overall conductivity [10]. (2) Reducing the interfacial resistance at polymer–ceramic contacts. Polymers (e.g., PEO and PVDF) conduct Li⁺ primarily through segmental chain motion, while ceramics rely on vacancy-mediated lattice diffusion [11]. At the interface, Li⁺ hopping across these heterogeneous phases creates energy barriers, and space-charge layers would further impede Li⁺ transfer by establishing ion concentration gradients [12–14]. As a result, many CSEs exhibit low Li^+^ transference numbers, often below 0.4 [15–19]. Various interfacial engineering approaches have been developed to mitigate these limitations. They include coatings with Li^+^-conductive layers such as lithium phosphate [20], ionic liquids [21], silane coupling agents like 3-isocyanotopropyltriethoxytsilane [22], 3-methacryloxypropyltrimethoxysilane [23], and oxygen-rich polymer interlayers. Among them, polymer interlayers are particularly promising [24–26]. Polydopamine (PDA) is a notable example due to its strong interfacial adhesion and chemical reactivity [27, 28]. When grafted onto ceramic surfaces, these interlayers offer multiple functional groups (i.e., catechol, carbonyl, and hydroxyl) capable of interacting with both the polymer matrix and lithium ions and enabling enhanced ion conductivity [29–31]. While PDA has shown promise in improving filler dispersion and interfacial compatibility, most studies focus on macroscopic effects. The role of PDA in promoting selective Li⁺ transport at the polymer–ceramic interface remains largely unexplored. Beyond improving physical contact, there is a critical need for molecular-level interfacial designs that facilitates directional Li⁺ transfer and inhibits anion migration.

**Fig. 1:**
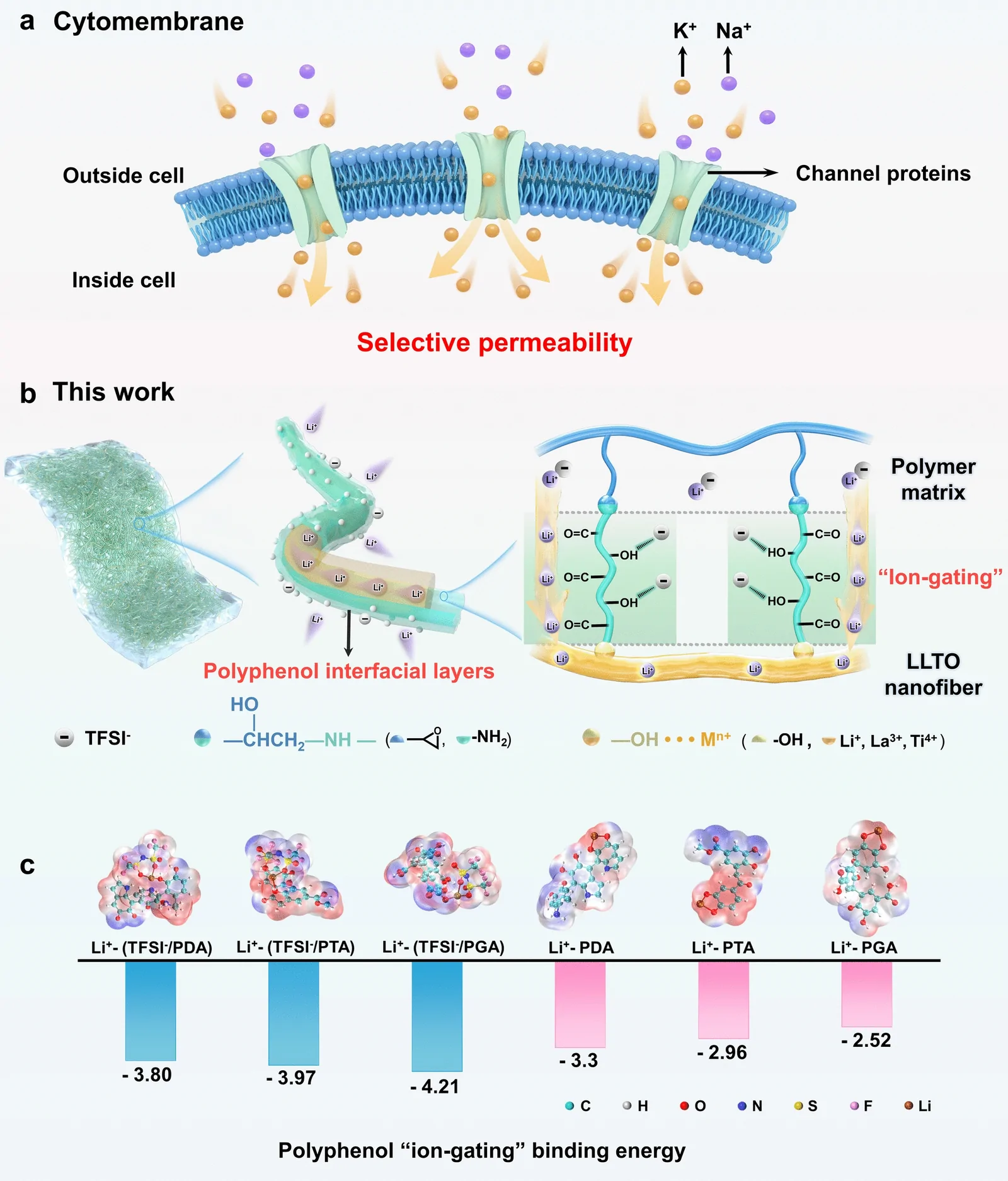
Design of bioinspired ion-selective interface and Density Functional Theory (DFT) calculations of polyphenol interface.** a** Schematic illustration of selective permeability through cytomembranes. **b** Illustration of the polyphenol-gated interfacing, highlighting the chemical binding and bionic “ion-gating” effect at the inorganic-polymer interface. **c** Binding energies and electrostatic potential calculations of Li^+^-TFSI^−^ complexes at PDA, PTA, and PGA interfaces, including Li^+^-C=O coordination strengths

In biological systems, the cytomembrane containing channel proteins can control the migration of ions (like Na^+^, K^+^, or Ca^2+^) in and out of the cell precisely (Fig. 1a). The narrow channels consisting of carbonyl groups form stable coordination with K^+^ and allow the passage, while restricting that of other ions [32]. Inspired by this natural principle, we introduce polyphenols including PDA, poly-tannic acid (PTA), and poly-gallic acid (PGA) to build Li⁺-selective transport channels across the polymer-ceramic interface (Fig. 1b). These interlayers serve as chemical gates in which carbonyl groups coordinate Li^+^ to lower interfacial energy barrier, and –OH and –NH groups immobilize bis(trifluoromethanesulfonyl)imide (TFSI^−^) anions via hydrogen bonding. This gating effect increases free Li⁺ concentration at the interface and enhances selective cross-phase ion transport compared to systems without polyphenol modification. In practical devices, Li||LiFePO_4_ cells with PDA-gated CSE exhibit excellent cycling stability over 1000 cycles at 5 C. The pouch cell achieves an initial capacity of 141.0 mAh g^−1^ with 82.9% retention after 400 cycles at 1 C. These results provide insight into regulating interfacial ion transport through molecular design of stable and high-rate solid-state lithium-metal batteries.

## Experimental Section

### Materials

Lithium nitrate (LiNO_3_), lanthanum nitrate hexahydrate (La(NO_3_)_3_.6H_2_O), titanium (IV) butoxide titanate, lithium bis (trifluoromethanesulphony) imide (LiTFSI), 2,2-azobis (2-methylpropionitrile) (AIBN), and poly (ethylene glycol) dimethyl ether (PEGDE, M_w_ = 500) were purchased from Adamas-beta, polyvinyl pyrrolidone (PVP, M_w_ = 1,300,000) and tannic acid (TA) were got from Sigma-Aldrich, and acetic acid, N,N-dimethylformamide (DMF), 1-meathyl-2-pyrrolidinone (NMP), poly (ethylene glycol) methyl ether acrylate (PEGMA, M_w_ = 480), polyethyleneimine (PEI, M_w_ = 400), dopamine hydrochloride (DA), gallic acid (GA), and glycidyl methacrylate (GMA) were purchased from Aladdin. Polyvinylidene difluoride (PVDF, HSV900) was bought from Dongguan west plastic trade Co., Ltd. Active materials LiFePO_4_ (LFP) and LiNi_0.8_Co_0.1_Mn_0.1_O_2_ (NCM811) were provided by Shenzhen Kejingstar (MTI) Technology Co., Ltd. And carbon black was purchased by Suzhou Sinero Technology Co., Ltd.

### Preparation of the La_0.56_Li_0.33_TiO_3_ (LLTO) Ceramic Nanofiber Electrolyte

The LLTO ceramic nanofiber electrolyte was prepared by electrospinning followed by high-temperature sintering method. Briefly, 0.262 g LiNO_3_, 2.425 g La(NO_3_)_3_.6H_2_O, and 3.403 g titanium (IV) butoxide titanate were added into 16 g DMF solvent with 16 wt% PVP and 13 wt% acetic acid. The mixture was stirred for 12 h to obtain a yellow transparent solution. The solution was then transferred into a 10 mL syringe and electrospun for 10 h to obtain the LLTO membrane precursor, named as p-LLTO. The electrospinning parameters were set as follows: the needle-to-collector distance was 15 cm, the injection speed was 0.08 mm min^−1^, and the applied voltage was 15 kV. The p-LLTO was then transferred to an alumina boat and subjected to a segmented sintering process: calcination at 600 °C for 1 h with a heating rate of 2 °C min^−1^, followed by annealing at 800 °C for 2 h in air to obtain the final LLTO ceramic nanofibers. Finally, the LLTO membranes were cut into 16-mm slice for battery assembly.

### Preparation of Polyphenol-Modified LLTO Ceramic Nanofiber Electrolytes

DA (2 mg mL^−^^1^) and PEI (2 mg mL^−^^1^) were dissolved in 40 mL Tris buffer (pH = 8.5). Then, the LLTO nanofiber membrane was immersed in the above solution and placed in the dark for 12 h at room temperature. The modified LLTO membrane, signed as PDA_2_@LLTO, was then rinsed 3 times with ultrapure water and dried in an oven at 80 °C for 24 h. Similarly, PDA_0.5_@LLTO and PDA_4_@LLTO were also prepared using the same method with the DA concentrations of 0.5 and 4 mg mL^−^^1^, respectively.

Additionally, PTA_2_@LLTO and PGA_2_@LLTO membranes were synthesized using the same method with reactive monomer of TA and GA.

### Preparation of Composite Solid-State Electrolytes

GMA monomers (1 g), PEGMA monomers (2 g), PEGDE (0.3 g), AIBN thermal initiator (0.03 g), and LiFTSI (0.66 g) were mixed and stirred for 2 h to obtain a homogeneous glycidyl polyether (GE) precursor solution. Subsequently, the precursor solution (60 μL) was dropped onto the prepared PDA_x_@LLTO membrane and polymerized at 65 °C for 3 h. The final composite solid-state electrolyte, donated as PDA_x_@LLTO/GE, was obtained after heating for additional 1 h at 100 °C to form the chemical bonding between the polyphenol layer and GE matrix. All steps and subsequent battery assembling were carried out in an argon-filled glovebox (O_2_ < 0.01 ppm, H_2_O < 0.01 ppm). PTA_2_@LLTO/GE and PGA_2_@LLTO/GE electrolytes were prepared using the same method.

### Preparation of the Cathodes

LFP, super P, and PVDF with the mass ratio of 7:2:1 was mixed in NMP to form a uniform slurry of 10% solid content. Then, the slurry was coated on a carbon-coated aluminum foil and dried in a vacuum oven at 80 °C for 2 days to completely remove the solvent. And the dried electrode was then cut into a 12 mm disk with a mass loading of about 1 ~ 1.4 mg cm^−^^2^. For the high LFP loading cathode, the areal loading of LFP was controlled at 5.0 mg cm^−^^2^. The NCM811 cathode was prepared by the same method using a mixture of NCM811, super P, and PVDF binder solution in NMP at a mass ratio of 8:1:1, and the corresponding active material loading was 1 mg cm^−^^2^.

### Materials Characterizations

The microstructure of the samples was characterized by field-emission electron microscope (FESEM, JSM 7500 F). Transmission electron microscope (TEM, Talos F200S) was used to analyze the morphology LLTO ceramic nanofibers before and after modifying with polyphenol layer, and energy-dispersive spectrometer (EDS) was used to map the surface element distribution. The crystal structure of LLTO was confirmed by X-ray diffraction (XRD, Bruker AXS D2 Phaser). Fourier transform infrared (FI-IR, Nicolet 6700) spectra and X-ray photoelectron spectroscopy (XPS, Thermo Scientific ESCALAB 250Xi equipped with Al, K_α_ X-ray source) spectra were conducted to characterize the chemical interactions between PDA and the LLTO nanofibers and polymer matrix. ^7^Li solid-state nuclear magnetic resonance (NMR) and temperature-variable FTIR spectra results were collected by Bruker Avance 400 and Nicolet iS50 FTIR to investigate the distribution state of Li^+^ in the CPEs and the Li^+^ transport behavior at the polyphenol interfaces. Raman spectroscopy was carried out with Renishaw in Via-Reflex (532 nm). The mechanical properties were tested using the tensile machine (UTM2103, Shenzhen suns technology CO., Ltd.). The thermodynamic behavior was studied by differential scanning calorimetry (DSC, PerkinElmer DSC4000). The thermogravimetric analysis (TGA, NETZSCH TG 209F1 Libra) was measured under N_2_ atmosphere to study the thermostability of the electrolytes.

### Electrochemical Measurements

All electrochemical properties were performed using CR2025 coin cells. A CHI760E electrochemical workstation was employed to measure the electrochemical impedance spectroscopy (EIS), electrochemical window, and chronoamperometry spectra. All the electrochemical properties of the electrolytes, such as galvanostatic discharge/charge profiles, long cycling stability, and rate capability, were conducted at 60 °C using a LAND test system.

The impedance of the electrolytes was measured by EIS test in a frequency range from 100 kHz to 0.1 Hz. And the ionic conductivity was obtained from the symmetric cell assembled with two stainless steels and calculated using the following equation:

1 $$\sigma = \frac{L}{R \times S}$$

where σ is ionic conductivity, *L* is the thickness of the electrolyte, *R* is the impedance, and *S* is the contact area between the electrolyte and the stainless steel.

The activation energy (*E*_*a*_) of the electrolytes was calculated using the Arrhenius equation:

2 $$\sigma {\text{ = Ae}}^{{\frac{{\text{ - Ea}}}{{{\mathrm{RT}}}}}}$$

where A is the pre-exponential factor, R is the molar gas constant (8.314 J mol⁻^1^ K⁻^1^), and T is the absolute temperature.

The Li^+^ transference number (*t*_Li_^+^) of electrolytes was measured using a symmetric Li||Li cell with a chronoamperometry test under a DC voltage amplitude of 10 mV at room temperature. And the impedances before and after polarization were also measured by EIS. The *t*_Li_^+^ can be calculated according to Eq. (3):

3 $$t_{{{\mathrm{Li}}}}^{ + } = \frac{{I_{s} {(}\Delta {\text{V - R}_{0}I_{0})}}}{{I_{0} {(}\Delta {\text{V - R}_{s}I_{s})}}}$$

where *I*_0_ and *I*_s_ are the initial and steady-state DC currents, *R*_0_ and *R*_*s*_ are the interfacial impedances before and after polarization, and ∆*V* is the applied voltage (10 mV).

The electrochemical window stability of the electrolytes was evaluated by LSV using a Li||SS half-cell at a scan rate of 0.1 mV s^−1^ at room temperature. Galvanostatic cycling tests were conducted on LFP||Li full cells within a voltage range of 2.4–4.2 V.

### Simulation Methods

#### Density Functional Theory (DFT) Calculations

DFT calculations and electrostatic potentials (ESP) were carried out by the Central Laboratory, School of Chemical and Material Engineering, Jiangnan University, to investigate the interactions between Li^+^, TFSI^−^, PDA, PTA, and PGA. All calculations were performed using the Gaussian 16 software package. The B3LYP functional was adopted for all calculations in combination with the D3BJ dispersion correction. In geometry optimization and frequency calculations, the 6-31G(d,p) basis set was used. The singlet point energy calculations were performed with a larger basis set combination, in which the ma-def2-TZVP basis set was used. The optimized structures were used to calculate the ESP and adsorption energies. The most negative regions in the ESP were identified as the adsorption sites for lithium-ion. Therefore, the binding energy (*E*(AB)_Binding_) was calculated using Eq. (4):

4 $$E({\mathrm{AB}})_{{{\mathrm{Binding}}\,}} = E({\mathrm{AB}}) - E(A) - E(B)$$

where *E*(AB), *E*(*A*), and *E*(*B*) represent the single-point energy of complex AB, component A, and component B, respectively.

#### Molecular Dynamics (MD) Simulations

MD simulations were performed using the GROMACS package to probe Li^+^ transport across the LLTO/polymer interface at the atomic level in LLTO/GE and PDA_2_@LLTO/GE systems. The initial configurations for the two systems were constructed using PACKMOL software. The parameter fitting was performed via the density functional theory (DFT) package Orca, using the B3LYP exchange–correlation functional supplemented with Grimme’s DFT-D3(BJ) empirical dispersion correction. For all species in this work, the RESP2(0.5) charges (combining gas-phase and implicit solvent (SMD model) liquid-phase charge distributions) were calculated using Orca and the Multiwfn package, and this non-bonded force field charge model was adopted for subsequent MD simulations.

The MD simulation was first performed in the NVT ensemble at 298 K with a 1 fs time step for 1 ns. Then, the equilibrium simulation was conducted in the NPT ensemble (1 bar, 298 K) in a cubic box (periodic boundary conditions applied in all directions) with a 1 fs time step for 2 ns. Finally, the production simulation was carried out in the NVT ensemble at 298 K with a 1 fs time step for 1 ns. Radial distribution functions (RDF, *g*_ij_(*r*)) and coordination numbers (CN, *N*_*i*_) are computed as:

5 $$ N_{i}  = 4\pi n_{j} \int\limits_{0}^{{R_{M} }} {g_{{ij}} } (r)r^{2} dr $$

where *R*_*M*_ corresponds to the distance of the first minimum after the first peak in the *g*_*ij*_(*r*). All the system visualizations of MD simulations are rendered by VMD software. The mean square displacement (MSD) of Li^+^ is calculated by the Einstein equation:

6 $${\mathrm{MSD}} = \, < \left[ {R(t)\, - \,R(0)} \right]^{2}>$$

in which *R*(*t*) and *R*(*0*) are the positions of the Li^+^ atoms at time t and 0. And the diffusion constant (*D*) is the slope of MSD versus time with a factor of 1/6:

7 $$D = \frac{1}{6}\mathop {\lim }\limits_{t \to \infty } \frac{{\mathrm{d}}}{{{\mathrm{d}}t}}{\mathrm{MSD}}$$

## Results and Discussion

### Bioinspired Ion-Selective Interfaces Design and Structural Characterizations of Polyphenol-Gated CSEs

As a molecular basis for “ion-gating” effect, we performed density functional theory (DFT) calculations to assess the interactions between lithium salts and polyphenol layers (Fig. 1c). The results show that TFSI^−^ anions form stable hydrogen bonds with –OH and –NH groups in all three polyphenols, as indicated by the negative binding energy (∆E) (Fig. S1). These anion-specific interactions immobilize TFSI^−^ at the interface and weaken its coordination with Li⁺. To quantify this effect, we examined ternary complexes consisting of Li^+^, TFSI^−^, and polyphenol molecules. Compared to the strong Li⁺-TFSI^−^ binding energy of – 6.39 eV (Fig. S2a), the ternary complexes Li⁺-TFSI^−^/PDA (– 3.80 eV), Li⁺-TFSI^−^/PTA (– 3.97 eV), and Li⁺-TFSI^−^/PGA (– 4.21 eV) display significantly reduced binding energies (Fig. 1c). This reduction suggests that polyphenol participates in the solvation structure and promotes lithium salt dissociation, thereby enriching the interfacial region with mobile Li⁺ ions. Electrostatic potential (ESP) analysis supports this observation. While Li^+^ exhibits a highly localized positive field potential when bound to TFSI^−^ alone (Fig. S2b), its ESP distribution becomes more diffuse in polyphenol-containing complexes. This shift indicates a weakened Li⁺–TFSI^−^ interaction and a higher local concentration of Li^+^ at the interface. Moreover, carbonyl groups in the polyphenol backbones provide additional coordination sites for Li^+^. The calculated ∆E of Li^+^ with these carbonyl groups are − 3.30 eV (PDA), − 2.52 eV (PGA), and − 2.96 eV (PTA). This suggests strong ion–dipole interactions that facilitate directional Li^+^ migration. Altogether, these theoretical findings verify that polyphenol interlayers can act as ion-selective gates to facilitate Li^+^ transport into the ceramic phase.

**Fig. 2:**
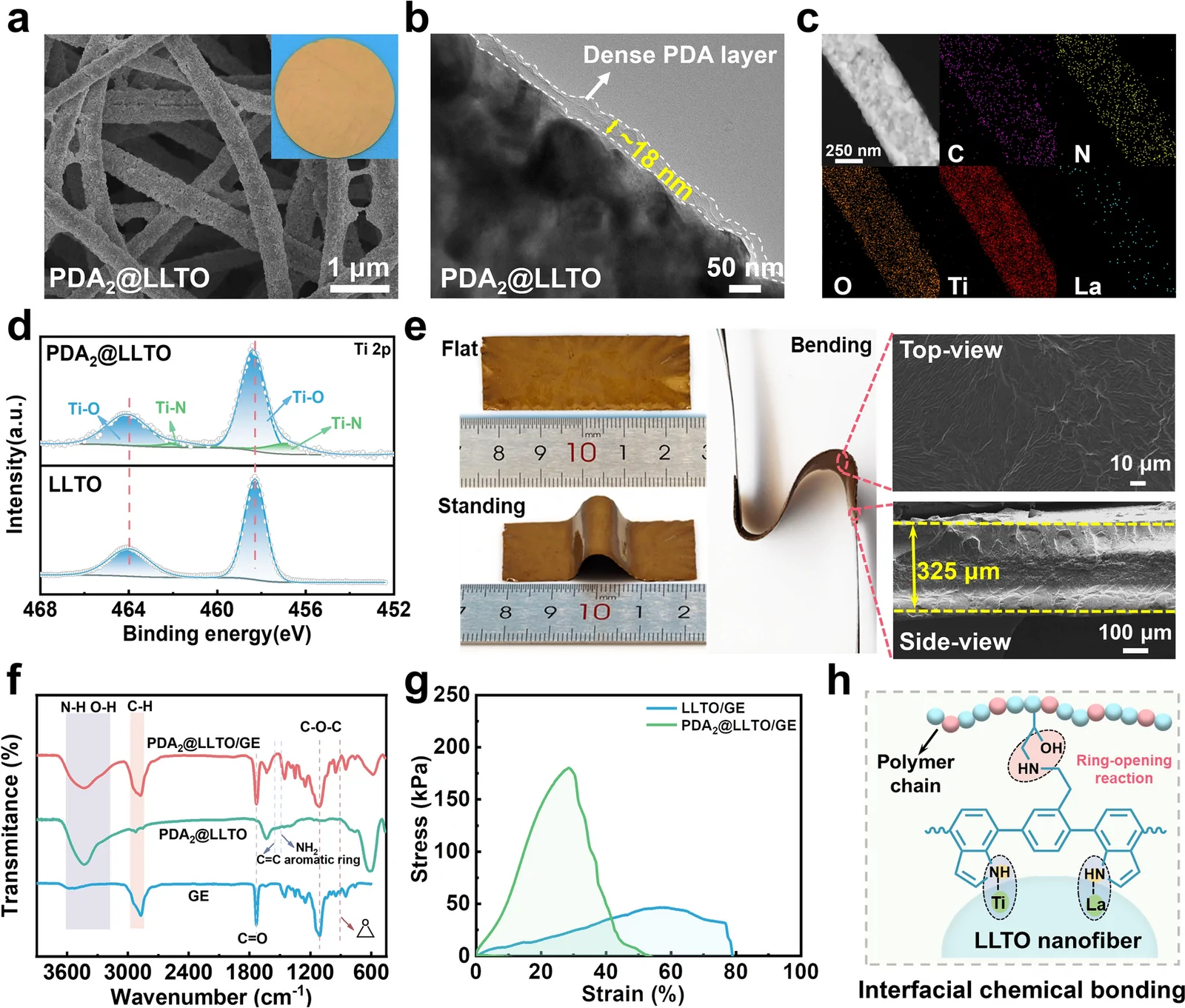
Synthesis and characterization of PDA_2_@LLTO/GE electrolyte.** a** SEM, **b** TME images, and **c** corresponding EDS elemental mappings of PDA_2_@LLTO nanofibers. **d** Ti 2*p* XPS spectra of LLTO and PDA_2_@LLTO membranes. **e** Photographs of PDA_2_@LLTO/GEs showing its flexibility, inset: top-view and side-view morphology. **f** FTIR spectra of GE, PDA_2_@LLTO, and PDA_2_@LLTO/GE electrolytes. **g** Tensile stress–strain curves of LLTO/GE and PDA_2_@LLTO/GE electrolytes. **h** Schematic of interfacial chemical bonding between the PDA interlayer, GE matrix, and LLTO nanofibers

We selected PDA-gated CSEs as a representative system to demonstrate our fabrication protocol which is illustrated in Fig. S3. A freestanding La_0.56_Li_0.33_TiO_3_ (LLTO) nanofiber membrane was first formed via electrospinning followed by solid-state sintering. The resulting LLTO nanofibers exhibit porous, grain-connected structures with a diameter of ~ 300 nm, as confirmed by scanning electron microscopy (SEM) and transmission electron microscope (TEM) (Figs. S4, S5). To introduce the “ion-gating” layer, a mussel-like- PDA coating was then deposited on the LLTO surface (diameter: 16 mm, thickness ~ 150 μm). The PDA-coated LLTO, denoted PDA_x_@LLTO (dopamine concentration x = 0.5, 2, and 4 mg mL^−^^1^) exhibits a uniform surface coverage, as visualized by SEM images compared to bare LLTO nanofibers (Figs. 2a andS6). TEM images (Figs. 2b and S7) reveal a conformal PDA coating on LLTO nanofibers with thicknesses of approximately 10, 18, and 29 nm for PDA_0.5_@LLTO, PDA_2_@LLTO, and PDA_4_@LLTO, respectively. Elemental mappings confirm the homogeneous distribution of C and N elements and verify the successful PDA functionalization (Fig. 2c). X-ray diffraction (XRD) patterns (Fig. S8) demonstrate that the crystalline structure of LLTO remains intact after PDA coating. X-ray photoelectron spectroscopy (XPS) analysis was carried out to investigate the chemical interactions at the interface. In the Ti 2*p* spectrum (Fig. 2d), PDA_2_@LLTO exhibits shifted Ti–O peaks along with new signals at 456.7 and 461.5 eV. This can be attributed to Ti–N bonds formed between Ti^4+^ in LLTO and amine groups in PDA. Additional evidence of La–N bond formation (Fig.  S9) supports the strong interfacial coordination between the PDA layer and the LLTO surface.

Subsequently, glycidyl polyether (GE) was infiltrated into the coated nanofiber scaffold via thermopolymerization of glycidyl methacrylate and poly(ethylene glycol) methyl ether acrylate. This flexible, integrated CSE membrane is denoted as PDA_x_@LLTO/GE. The PDA_2_@LLTO/GE membrane exhibits a uniform thickness of ~ 325 μm and excellent flexibility (Fig. 2e). Thermogravimetric analysis (Fig. S10) indicates that the inorganic LLTO content in the final membrane is approximately 20 wt%, which is significantly higher than the typical filler limit (~ 10 wt%) in conventional inorganic nanoparticles (such as LLZTO)-based CSEs [33]. This increased loading originates from the continuous fibrous framework, which effectively mitigates filler agglomeration and ensures uniform phase dispersion within the polymer matrix. Fourier transform infrared (FTIR) spectroscopy (Fig. 2f) exhibits the weakened epoxy peak at 910 cm^−^^1^ in PDA_2_@LLTO/GE compared to GE and reveals the ring-opening reaction of GE’s epoxy groups induced by amino groups in PDA. The enhanced C–O–C stretching peak (~ 1108 cm^−^^1^) is in accord with structural rearrangements in the GE network upon interaction with PDA. Altogether, these results indicate strong interaction between PDA and the polymer matrix. Differential scanning calorimetry (DSC) curves show a slightly increased glass transition temperature (T_g_) and reflect an altered polymer chain mobility due to interfacial interactions (Fig. S11). Adhesion tests demonstrate strong contact with Li metal (80 N m^−^^1^ adhesion energy, Fig. S12), and the tensile strength of PDA_2_@LLTO/GE is four times higher than that of unmodified the LLTO/GE membrane (Fig. 2g). These results confirm the successful construction of a homogeneous, chemically integrated PDA interlayer that bridges polymer and ceramic phases (Fig. 2h), which reinforces the structural integrity of the composite electrolyte. Besides, endowed with abundant functional groups, this structurally robust PDA interlayer underpins the subsequent formation of the ion-selective interface, enabling enhanced solid-state lithium transport. To demonstrate the general applicability of the polyphenol-gated interfacial design, PTA_2_@LLTO and PGA_2_@LLTO were also synthesized using the same method. SEM analysis (Fig. S13) confirms successful polymer coating and supports the structural foundation for Li⁺-selective interface across different polyphenol systems.

### Exploring the Ion Transport Properties and “Ion-Gating” Mechanism in Polyphenol-Gated CSEs

**Fig. 3:**
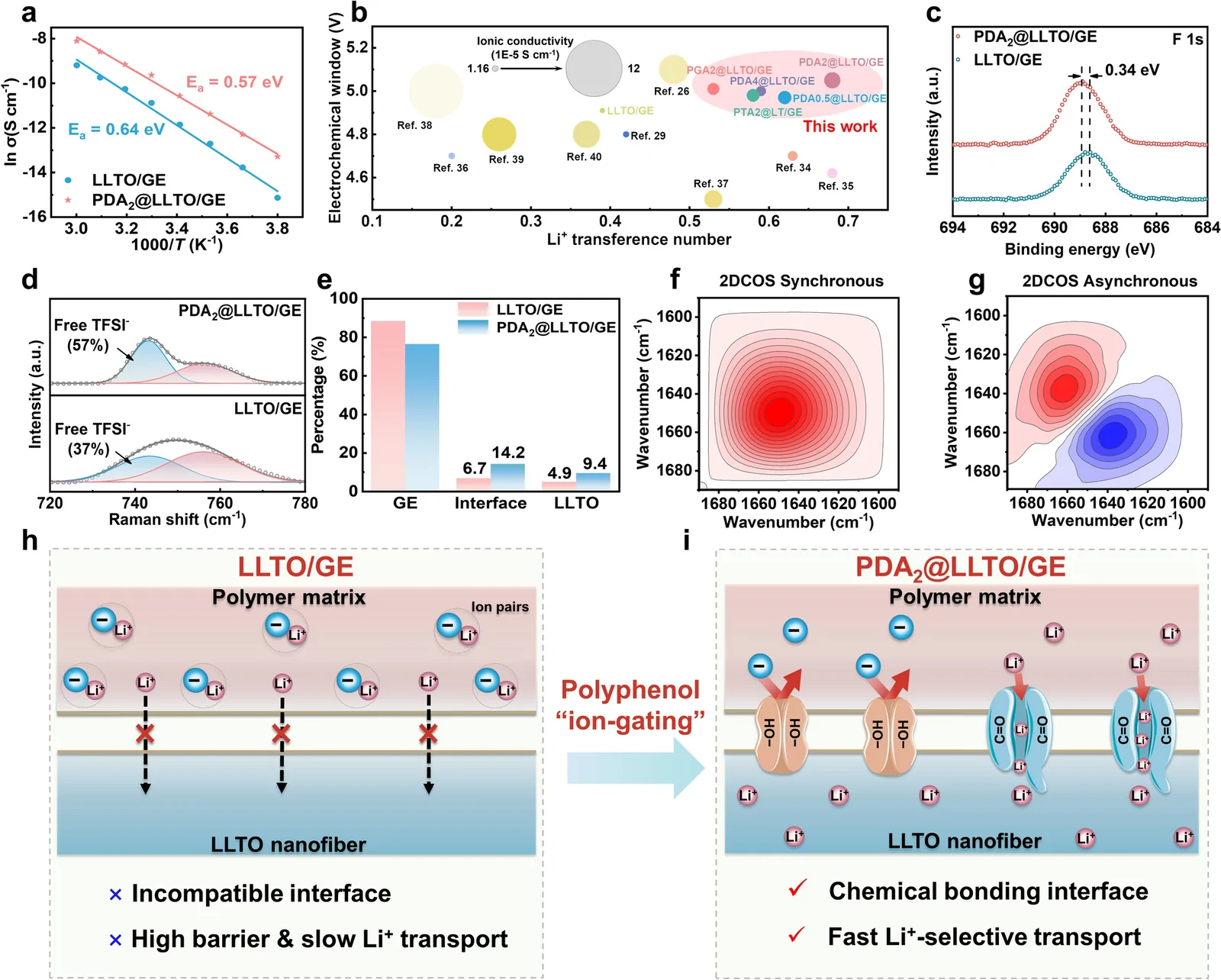
Electrochemical performance of PDA_2_@LLTO/GE electrolyte and Li^+^-transport mechanism at polyphenol interface.** a** Arrhenius plots of ion conductivities (σ) of LLTO/GE and PDA_2_@LLTO/GE electrolytes. **b** Comparison of σ, t_Li_^+^ and electrochemical windows for various reported CSEs at room temperature (The circle size represents σ). **c** F 1*s* XPS spectra, and **d** Raman spectra of LLTO/GE and PDA_2_@LLTO/GE electrolytes. **e** Quantitative Li^+^ distribution in LLTO/GE and PDA_2_@LLTO/GE electrolytes derived from solid-state ^7^Li NMR spectra. **f** Synchronous, and **g** asynchronous 2DCOS spectra of PDA_2_@LLTO/GE electrolyte (red: positive; blue: negative intensities). **h, i** Schematic illustration of Li^+^ transport pathways at the organic–inorganic interface in LLTO/GE and PDA_2_@LLTO/GE, respectively

We next evaluated the ion transport performance of polyphenol-gated CSEs. According to electrochemical impedance spectroscopy (EIS, Fig. S14), the PDA_2_@LLTO/GE electrolyte delivers a high ion conductivity (σ) of 3.01 × 10^−^^4^ S cm^−^^1^ at 60 °C, more than four times higher than that of LLTO/GE (7.12 × 10^−^^5^ S cm^−^^1^). This improvement was further analyzed through temperature (*T*)-dependent conductivity measurements. From the Arrhenius plots (σ vs. T, − 10 to 60 °C), the activation energy (E_a_) for Li⁺ transport in PDA_2_@LLTO/GE was calculated to be 0.57 eV, lower than the value of 0.64 eV for LLTO/GE (Fig. 3a): there is enhanced Li⁺ migration across the interface in the PDA-gated system. Additionally, the influence of PDA thickness on the ionic conductivity was further investigated. As shown in Fig. S15, a thin PDA layer is discontinuous, failing to fully eliminate the interfacial voids at the GE matrix and LLTO filler. Notably, an over-thick layer will deteriorate the Li^+^ migration due to the prolonged interfacial ion transport pathway caused by the intrinsically low ion conduction of PDA barrier. Therefore, the optimized PDA_2_@LLTO/GE achieves highest room-temperature ionic conductivity of 3.57 × 10^−^^5^ S cm^−^^1^. In terms of electrochemical stability, linear sweep voltammetry (LSV) shows that the PDA_2_@LLTO/GE electrolyte possesses an extended electrochemical window of up to 5.05 V versus Li/Li^+^, compared to that of LLTO/GE (Fig. S16). This broader voltage range indicates improved oxidative stability and suggests better compatibility at the electrolyte–electrode interface. Moreover, the Li^+^ transference number (t_Li_^+^) increases from 0.39 in LLTO/GE to 0.68 in PDA_2_@LLTO/GE at 60 °C. This can be attributed to effective immobilization of TFSI⁻ by the polar groups of PDA (Fig. S17). We evaluated two additional polyphenol-modified CSEs, namely PTA_2_@LLTO/GE and PGA_2_@LLTO/GE, to verify the general applicability. These systems achieve improved room-temperature ion conductivities of 2.79 × 10^−^^5^ and 2.61 × 10^−^^5^ S cm^−^^1^ as compared to that of LLTO/GE (1.17 × 10^−^^5^ S cm^−^^1^). Their t_Li_^+^ also increases to 0.58 (PTA) and 0.53 (PGA), respectively (Figs.  S18 and S19). The observed differences in ionic conductivity and t_Li_^+^ among the three polyphenol interfaces likely stem from the limited functional groups within PTA (–OH) and PGA (–COOH), whereas the PDA features more abundant functional polar groups (–OH, –NH), thereby enabling the PDA_2_@LLTO/GE to deliver the optimal ion transport kinetics. Besides, these values compare favorably with previously reported polymer-ceramic CSE systems (Fig. 3b) such as Li_1.3_Al_0.3_Ti_1.7_(PO_4_)_3_/poly (vinylidene fluoride-hexa-fluoropropylene) (LATP/PVDF-HFP) and β-cyclodextrin modified LLZTO/PEO that typically display small t_Li_^+^ (< 0.5), lower conductivity, and limited stability [26, 29, 34–40].

To gain insights into Li^+^ transport enhancement across at the polyphenol-gated interface, we employed multiple analytic techniques. XPS was performed to assess the interaction between Li^+^ and TFSI^−^ in the CSEs (Fig. 3c). The F 1*s* peak of TFSI^−^ shifts from 688.64 eV in LLTO/GE to 688.90 eV in PDA_2_@LLTO/GE, indicating improved interaction between TFSI^−^ and the polar groups in PDA, which weaken Li^+^-TFSI^−^ binding and promote salt dissociation. Raman analysis supports this result (Fig. 3d). The free TFSI^−^ peak at 743 cm^−^^1^ becomes more intense in PDA_2_@LLTO/GE, while the undissociated in LLTO/GE shifts slightly [41]. The fraction of free TFSI^−^ increases by ~ 20%, implying higher Li⁺ availability for conduction. Solid-state ^7^Li magic-angle spinning nuclear magnetic resonance (MAS NMR) reveals changes in Li^+^ distribution in different environments. In pure LLTO nanofibers, broad ^7^Li signals appear at 1.23 and 2.95 ppm. In contrast, GE shows narrowed peaks at 0.64 and 0.97 ppm (Fig. S20a), suggesting restricted Li^+^ mobility in the polymer matrix. In LLTO/GE, deconvoluted peaks disclose Li^+^ populations in the GE matrix (88.4%), LLTO nanofibers (4.9%), and GE-LLTO interface (6.7%). In PDA_2_@LLTO/GE, the Li^+^ content at the inorganic phase and PDA-LLTO interface increases to 9.4% and 14.2%, respectively **(**Figs. 3e and S20b, c). This indicates enhanced Li^+^ exchange across the interface. Accordingly, the PDA interlayer effectively promotes interfacial Li^+^ transport and strengthens the contribution of the 3D ceramic network to overall ion conductivity.

To study the interaction between Li^+^ and carbonyl functional groups at different temperatures, we recorded temperature-dependent FTIR spectra. In PDA_2_@LLTO/GE, the C=O‧‧‧H band at 1648 cm^−^^1^ and C=O‧‧‧Li^+^ at 1629 cm^−^^1^ both shift to lower wavenumbers as the temperature increases from 30 to 100 °C, accompanied by a decrease in peak intensity (Fig. S21a) [42]. This behavior suggests enhanced Li^+^-carbonyl interaction at elevated temperatures, indicating stronger coordination upon heating [43]. Two-dimensional correlation spectroscopy (2DCOS), including synchronous and asynchronous spectra, was employed to analyze the thermal changes of hydrogen-binding and lithium-binding carbonyl (Fig. 3f, g). According to Noda’s rule and the temperature-variable FTIR results [44, 45], three characteristic carbonyl modes were identified: 1668 cm^−^^1^ (C=O in dipole–dipole, C=O‧‧‧C = O), 1648 cm^−^^1^ (C=O in hydrogen bonding, C=O‧‧‧H), and 1629 cm^−^^1^ (C=O in Li^+^ coordination, C=O‧‧‧Li^+^), as summarized in Table S1. The order of these modes under thermal excitation reveals a strong C=O‧‧‧Li^+^ interaction (Fig. S23), which supports its key role in facilitating Li^+^ conduction. Compared to the poorly matched interface in the LLTO/GE case (Fig. 3h), the PDA interlayer achieves an “ion-gating” effect that promotes fast, Li^+^-selective transport across the organic–inorganic interface (Fig. 3i). Similar 2DCOS trends in PTA_2_@LLTO/GE and PGA_2_@LLTO/GE confirm the carbonyl-mediated Li^+^ transport as a key mechanism in polyphenol-gated CSEs (Figs. S21b, c and S22).

**Fig. 4:**
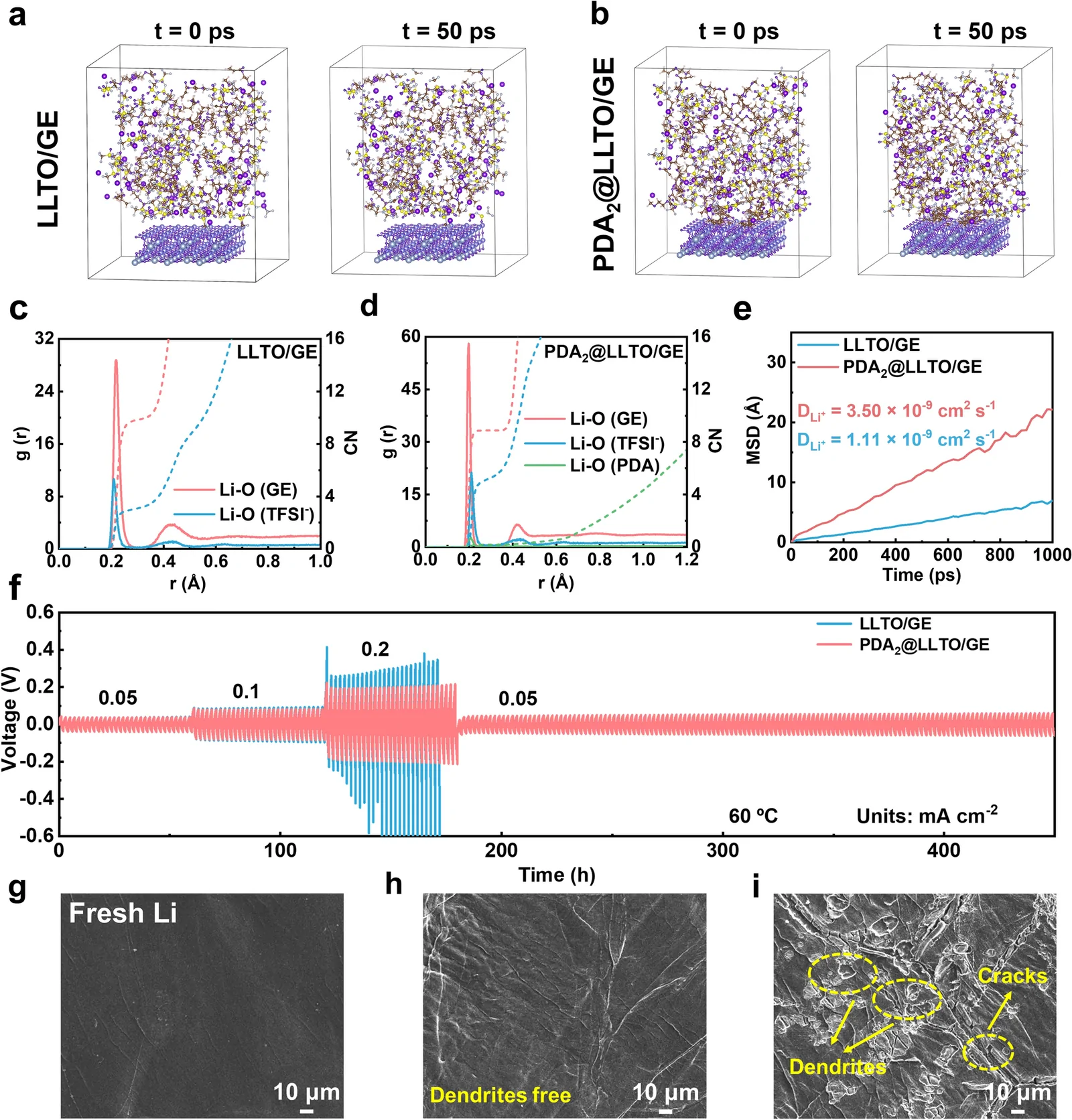
Molecular dynamics (MD) simulation and Li||Li symmetric cell performance of PDA_2_@LLTO/GE electrolyte. MD snapshots of **a** LLTO/GE and **b** PDA_2_@LLTO/GE at different time intervals. RDFs (solid line) and coordination numbers (dashed line) of Li–O pairs in **c** LLTO/GE and **d** PDA_2_@LLTO/GE. **e** Mean squared displacement (MSD) profiles of Li^+^ in LLTO/GE and PDA_2_@LLTO/GE. **f** Rate performance of symmetric cells using two electrolytes at different current density at 60 °C. SEM images of **g** fresh Li foil, and 100-cycled Li anode at 0.1 mA cm⁻^2^ in **h** Li| PDA_2_@LLTO/GE |Li and **i** Li| LLTO/GE |Li symmetric batteries

Molecular dynamic (MD) simulations were performed to visualize ion solvation environments. In the LLTO/GE system, time-resolved snapshots (Fig. 4a) show that Li^+^ ions remain closely coordinated with TFSI^−^ anions which indicates limited dissociation. In contrast, PDA_2_@LLTO/GE exhibits a disrupted Li^+^–TFSI^−^coordination structure and a higher concentration of free Li^+^ close to the interface (Fig. 4b). This observation is supported by an analysis of radial distribution functions (RDF). In both systems, the Li^+^–O (TFSI^−^) peak appears at 0.21 Å, reflecting initial ion pairing. However, the Li^+^–O (GE) peak shifts from 0.22 Å in LLTO/GE (Fig. 4c) to 0.20 Å in PDA_2_@LLTO/GE (Fig. 4d), along with an increase in intensity. This observation suggests stronger Li^+^ interactions with the polymer matrix and reduced association with TFSI^−^ in PDA_2_@LLTO/GE. In addition, a new peak at 0.21 Å, corresponding to Li⁺–O (PDA), confirms that the PDA interlayer contributes directly to Li^+^ coordination in an additional transport mechanism: the PDA layer introduces C=O groups that coordinate with Li⁺. At the same time, –OH and –NH groups stabilize TFSI^–^ through hydrogen bonding. Together, these interactions promote lithium salt dissociation and create additional pathways for Li⁺ migration. Mean-square displacement (MSD) analysis confirms the enhanced ion mobility. Li^+^ diffusion coefficient in PDA_2_@LLTO/GE reaches 3.50 × 10^−^^9^ cm^2^ S^−^^1^, significantly higher than 1.11 × 10^−^^9^ cm^2^ S^−^^1^ in LLTO/GE (Fig. 4e).

### Electrochemical Stability of the Li Symmetric Cells

Li symmetric batteries were assembled to assess the performance of PDA_2_@LLTO/GE electrolyte in the long Li plating/stripping process. The Li| PDA_2_@LLTO/GE |Li cell exhibits stable cycling for over 1600 h at 0.1 mA cm^−^^2^ with a low and consistent polarization voltage below 200 mV (Fig. S24). In contrast, the Li| LLTO/GE |Li cell shows a rapid increase in overpotential and fails after 350 h. This is likely due to severe side reactions stemming from poor interfacial ion transport. Furthermore, the lithium dendrite suppression capability was also evaluated. The PDA_2_@LLTO/GE endows the symmetric cell to reach a critical current density (CCD) of 0.6 mA cm^−^^2^, while the Li| LLTO/GE |Li cell exhibits a much lower value of 0.3 mA cm^−^^2^ (Fig. S25). Therefore, the PDA-modified CSE displays enhanced dendrite suppression under high-current density, which contributes to the stable and long-term cycling performance. The reversible rate capability was further evaluated by increasing the current density from 0.05 to 0.2 mA cm^−^^2^ (Fig. 4f). The Li| PDA_2_@LLTO/GE |Li cell maintains a stable polarization voltage across all current densities and shows a reversible behavior when the current returns to 0.05 mA cm^−^^2^. In comparison, the LLTO/GE-based cell exhibits severe voltage fluctuations under 0.2 mA cm^−^^2^. Throughout the tests, PDA_2_@LLTO/GE-based cell consistently exhibits lower polarization than LLTO/GE-based cell at all current densities (Fig. S26). This confirms enhanced ion transport enabled by the PDA-gated ion-selective interface. SEM images of cycled Li anodes show distinct surface differences. The Li anode with PDA_2_@LLTO/GE remains flat and smooth, with no visible dendrite formation after 100 cycles (Fig. 4g, h), whereas obvious cracks and dendrites are observed for the LLTO/GE system (Fig. 4i). These results underscore the power of a PDA-gated interface in promoting uniform Li⁺ flux and suppressing dendrite growth through fast cross-phase conduction pathways.

**Fig. 5:**
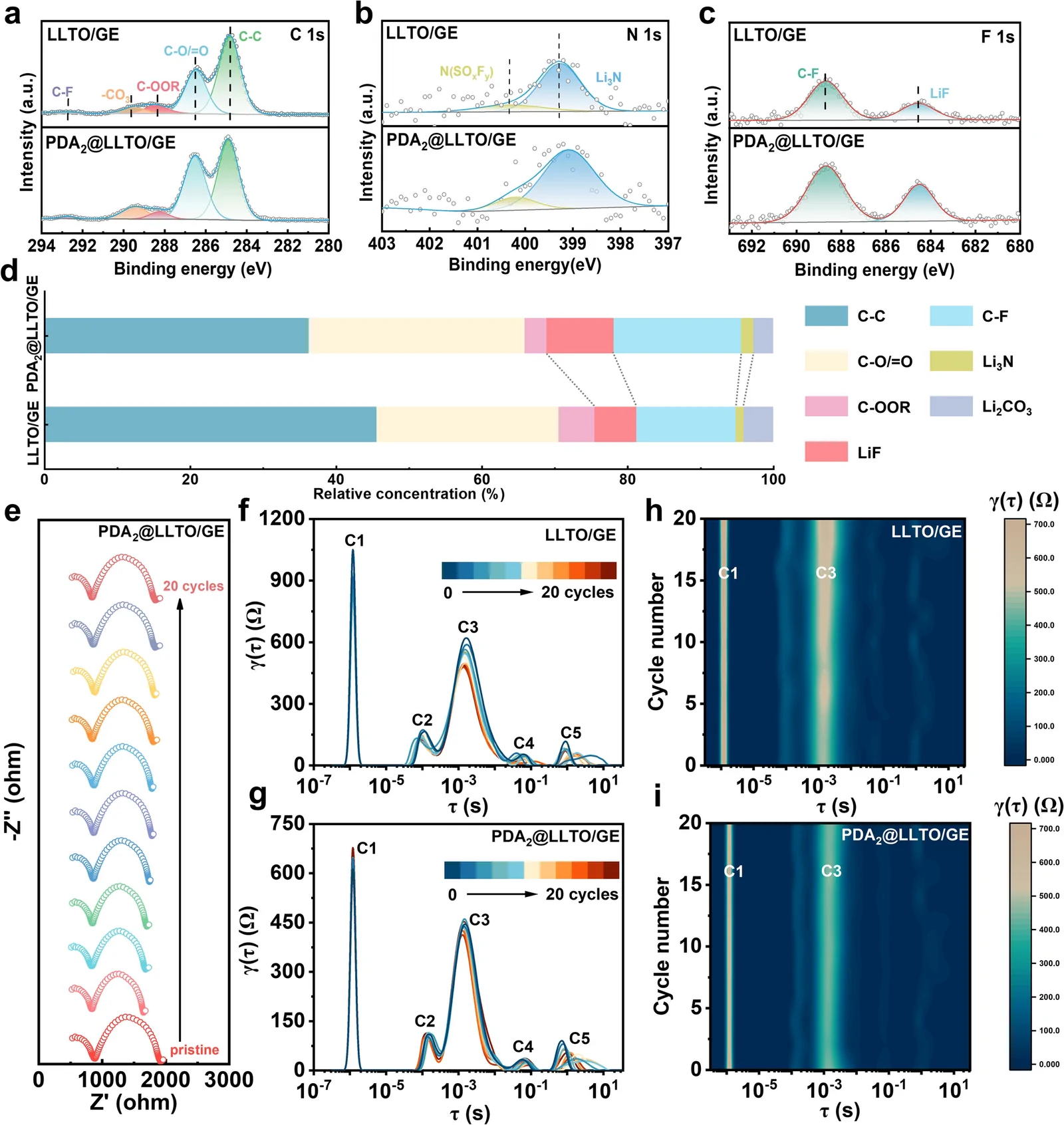
Electrolyte–electrode interface electrochemical properties.** a-c** XPS spectra of the Li surface after 20 cycles with LLTO/GE and PDA_2_@LLTO/GE electrolytes: **a** C 1*s*, **b** N 1*s,* and **c** F 1*s*. **d** Quantitative analysis of chemical components in the SEI layer for LLTO/GE and PDA_2_@LLTO/GE. **e** EIS plots of Li | PDA_2_@LLTO/GE | Li cell after different cycles. DRT analysis of **f, h** Li | LLTO/GE | Li and **g, i** Li | PDA_2_@LLTO/GE | Li cells after various cycles at 0.1 mA cm^−^^2^

The influence of PDA_2_@LLTO/GE on the formation of the solid electrolyte interphase (SEI) plays a critical role in governing Li deposition behavior and overall battery performance. The C 1*s* XPS spectra show stronger signals for C–C (284.8 eV) and C=O (286.5 eV) in PDA_2_@LLTO/GE than in LLTO/GE (Fig. 5a) which is evidence of a more flexible, organo-rich SEI layer that suppresses Li corrosion [46]. The S 2*p* spectra exhibit two characteristic peaks at 170.0 and 168.7 eV, which can be assigned to sulfate groups from LiTFSI decomposition (Fig. S27a). This decomposition contributes to an electrically insulating layer. Stronger signals of Li_3_N (Fig. 5b) and LiF (Fig. 5c) are observed in PDA_2_@LLTO/GE which are known to facilitate Li^+^ transport across CSE-Li anode interface (Fig. S27b) [47]. Quantitative analysis confirms a reduced proportion of poorly conductive Li_2_CO_3_ and increased content of LiF and Li_3_N in PDA_2_@LLTO/GE compared to LLTO/GE (Fig. 5d). Furthermore, to evaluate the cycling stability of the PDA_2_@LLTO/GE electrolyte, the depth-resolved XPS spectra were performed to investigate the SEI compositions on Li anodes after 100 cycles at a higher density current of 0.2 mA cm^−^^2^. As shown in Fig. S28a, with the increase of etching time, the signals of organic components decrease slightly at 30 s and then maintain nearly constant throughout the subsequent depth profile. This stable distribution enables the formation of a flexible physical barrier, which can effectively suppress persistent side reactions between the electrolyte and Li anode. Meanwhile, the elevated LiF content in Fig. S28b is conducive to achieving the uniform lithium deposition. And no distinct –OH signals are detected in the O 1*s* spectra (Fig. S28c), verifying the interfacial stability of the PDA layer throughout cycling. These analyses demonstrate, again, that the PDA-gated interface facilitates the formation of a favorable SEI composed of flexible organic species and highly Li⁺-conductive inorganic components. This interface ensures long-term, dendrite-free lithium deposition and stripping.

To gain electrochemical insight into interfacial stability, the EIS was conducted on symmetric Li cells over multiple cycles. The initial impedance of LLTO/GE is significantly higher than that of PDA_2_@LLTO/GE (Figs. 5e and S29). Moreover, the Li| LLTO/GE |Li cell exhibits large impedance fluctuations during 20 cycles at 0.1 mA cm^−^^2^. In contrast, the PDA_2_@LLTO/GE-based cell maintains a lower and more stable impedance. To correlate the impedance response with specific interfacial processes, we analyzed the distribution of relaxation times (DRT). Three main contributions were identified (Fig. 5f, g): C1 at the high-frequency region (τ ≈ 10^−^^6^ s), corresponding to bulk resistance (R_b_); C2 and C3 at the mid-frequency regions (τ ≈ 10^−^^4^ to 10^−^^3^ s), assigned to SEI impedance (R_SEI_); C4 and C5 at the low-frequency regions (τ ≈ 10^−^^2^ to 10^−^^1^ s), related to charge transfer impedance (R_ct_). Compared to the Li| LLTO/GE |Li cell, PDA_2_@LLTO/GE-based cell maintains low and stable R_SEI_ components (C2, C3) over 20 cycles (Fig. 5h, i), reflecting a robust and stable SEI layer. This stability supports consistent Li^+^ transport and aligns with the steady overpotential observed during long-term cycling.

### Electrochemical Performance of Polyphenol-Gated CSEs in Li||LFP Full Battery

**Fig. 6:**
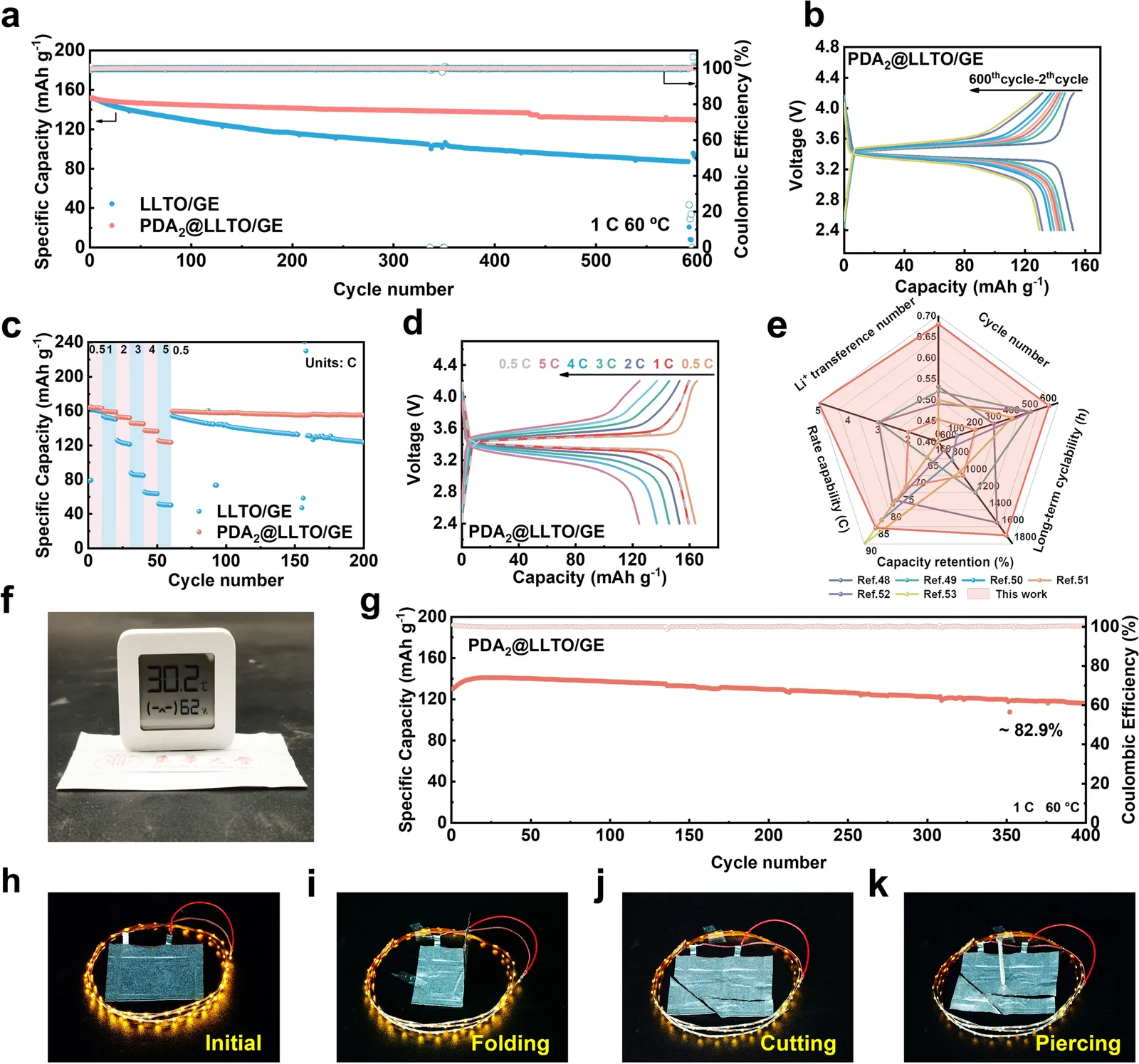
Cycling performance of PDA_2_@LLTO/GE electrolyte in LiFePO_4_||Li full cells at 60 °C.** a** Long-term cycling performance of LFP| LLTO/GE |Li and LFP| PDA_2_@LLTO/GE |Li full batteries at 1 C and 60 °C. **b** Charge/discharge profiles of LFP| PDA_2_@LLTO/GE |Li cell. **c** Rate performance of full batteries. **d** Charge/discharge profiles of LFP| PDA_2_@LLTO/GE |Li cell at 0.5—5 C. **e** Radar chart comparing electrochemical properties of different CSEs. **f** Digital image of LFP| PDA_2_@LLTO/GE |Li pouch cell powering a hygrothermograph. **g** Long-term cycling stability of pouch cell fabricated with PDA_2_@LLTO/GE at 1 C. **h–k** Safety tests of the pouch cell under folding, cutting, and piercing

We then evaluated the performance of full batteries assembled with LiFeO_4_ (LFP) cathode and PDA@LLTO/GE electrolyte at 60 °C. The LFP| PDA_2_@LLTO/GE |Li cell delivers a high initial specific capacity of 151.6 mAh g^−^^1^ and retains 85.5% of its capacity after 600 cycles at 1 C, with Coulombic efficiency (CE) consistently above 99.5% (Fig. 6a). In contrast, the LFP| LLTO/GE |Li cell suffers from rapid capacity decay, with only 90 mAh g^−^^1^ remaining after the same period. Galvanostatic charge/discharge curves demonstrate stable cycling and low polarization for LFP| PDA_2_@LLTO/GE |Li cells, with a capacity degradation rate of just 3.65% per cycle (Fig. 6b). Other PDA_x_@LLTO/GE also outperform LLTO/GE electrolyte (Fig. S30). Besides, even assembled with a high LFP loading of 5 mg cm^−^^2^, the PDA_2_@LLTO/GE still retains a superior discharge capacity of 135.1 mAh g^−^^1^ with 84.7% capacity retention after 100 cycles at 1 C, while the capacity of LFP| LLTO/GE |Li has declined to 76.5 mAh g^−^^1^ (Fig. S31). Under high-rate conditions, LFP| PDA_2_@LLTO/GE |Li cell achieves 125.7 mAh g^−^^1^ at 5 C, significantly higher than that of LLTO/GE cell (51.9 mAh g^−^^1^) (Fig. 6c). This cell also exhibits lower polarization across various current densities (Figs. 6d and S32) and even after 1000 cycles at 5 C can sustain 80.7 mAh g^−^^1^. By contrast, the LFP| LLTO/GE |Li cell declines from 93.0 mAh g^−^^1^ to just 43.2 mAh g^−^^1^ (Fig. S33a). Full cells coupled with PTA_2_@LLTO/GE and PGA_2_@LLTO/GE electrolytes also show high capacity retentions of 59% and 54%, respectively, after 1000 cycles (Fig. S33b). Compared with state-of-the-art CSEs such as silane coupling agents grafted LLZO/PEO and lithium phosphate modified LLZTO/PVDF [48–53], PDA_2_@LLTO/GE electrolyte demonstrates superior electrochemical performance and extended life span (Fig. 6e). To further validate the high-voltage stability of PDA_2_@LLTO/GE, NCM811||Li full cells were also assembled. As shown in Fig. S34, owing to the boosted ion conductivity and widened electrochemical window of PDA_2_@LLTO/GE, the resultant full cell delivers a high discharge capacity of 177.3 mAh g^−^^1^ and CE of 97.5% at the first cycle at 1 C. In contrast, the NCM811|LLTO/GE|Li only achieves an initial specific capacity of 153.1 mAh g^−^^1^ with a low CE of 93.2%. After 100 cycles, the unmodified LLTO/GE-based cell suffers severe capacity decay with a residual capacity of merely 74.9 mAh g^−^^1^, whereas the cell with PDA_2_@LLTO/GE maintains a high capacity of 100.5 mAh g⁻^1^ accompanied by stable voltage polarization throughout cycling. Additionally, the composition of the cathode electrolyte interphase (CEI) layer formed on the NCM811 cathode after 20 cycles at 1 C was investigated via XPS spectra. As illustrated in Fig. S35a, in PDA_2_@LLTO/GE, the organic component displays decreased signals after etching for 60 s, while the inorganic components (LiF and Li_2_O) show a significant enhancement at the inner region. They are well-recognized for the low-energy barriers for Li^+^ transport and can reduce the side reactions to stabilize the electrode–electrolyte interface (Fig. S35b, c). Therefore, the optimized CEI layer featuring with flexible organic outer layer and a robust LiF/Li_2_O-enriched inorganic inner layer ensures fast Li^+^ conduction and interfacial stability to achieve superior high-voltage durability and cycling performance. On the contrary, the sluggish ion transport in the LLTO/GE-based sample impairs the interfacial stability, which forms the CEI with more organic content derived from the decomposition of the polymer matrix (Fig. S35d) and insufficient LiF/Li_2_O (Fig. S35e, f), thus triggering intensive voltage polarization and rapid capacity degradation.

To validate the practical applicability, a single layer pouch battery with PDA_2_@LLTO/GE electrolyte was prepared. It exhibits a standard open-circuit voltage of 3.4 V and successfully powers an electronic console (Fig. 6f). This battery offers a high discharge capacity of 141.0 mAh g^−^^1^ with 82.9% retention after 400 cycles at 1 C (Fig. 6g). Impressively, the pouch battery continues to operate under harsh conditions such as folding, cutting, and puncturing without leakage or explosion (Fig. 6h-k). These findings demonstrate that the polyphenol-gated interface enables efficient cross-phase Li⁺ transport and interfacial stability, offering a compelling route toward next-generation solid-state lithium-metal batteries.

## Conclusions

In summary, we developed a polyphenol-gated CSE that mimics the cytomembrane consisting of channel proteins to suppress TFSI^−^ mobility and boosts Li^+^-selective transport across the polymer-ceramic interface. Compared to conventional CSEs, the optimized PDA_2_@LLTO/GE demonstrated a higher ionic conductivity of 3.01 × 10^−^^4^ S cm^−^^1^ at 60 °C and transference number of 0.68. As a result, PDA_2_@LLTO/GE enabled the stable cycling of the Li symmetric cell for over 1600 h and a high discharge capacity of 131 mAh g^−^^1^ for the LFP full cell even at 5 C. Furthermore, the pouch cell coupled with PDA_2_@LLTO/GE exhibited excellent cycling performance with 82.9% retention after 400 cycles at 1 C. However, their long-term stability under thermal and electrochemical stress still requires further investigation. Expanding the molecular design to a broader range of robust polyphenols may enhance interfacial durability and optimize performance. Moreover, applying this concept to electrolyte–electrode interfaces could help to mitigate polarization and improve the high-rate cycling stability and compatibility between CSEs and high-voltage cathodes. Overall, this work establishes a design concept for selective ion conduction across polymer-ceramic interfaces and provides new opportunities for the development of safer, longer-lasting solid-state lithium-metal batteries.

## Supplementary Information

Below is the link to the electronic supplementary material.

Supplementary file1 (DOCX 7307 kb)
