# Supplementary material for: Polyphenol-Gated Composite Electrolytes with Enhanced Cross-Phase Lithium-Ion Transport for Solid-State Lithium Batteries
Source: Nanomicro Lett. 2026 Mar 10;18:279. doi: 10.1007/s40820-026-02127-6 (PMC12976337; doi:10.1007/s40820-026-02127-6)
Supplement: Supplementary file 1 — Supplementary file1 (DOCX 7307 kb) [file 40820_2026_2127_MOESM1_ESM.docx]

Supporting Information for

**Polyphenol-Gated Composite Electrolytes with Enhanced Cross-Phase Lithium-Ion Transport for Solid-State Lithium Batteries**

Xiaoxiao Li^1^, Minqiang Jiang^1^, Kai Chen^1^, Zhixiang Cai^1^, Yingxin Zhang^1^, Jiamei Luo^1^, Lei Hou^2^, Yazhou Zhou^3^, Chao Zhang^1^, Hui Zhang^1^*, Feili Lai^4,5^*, Yue-E Miao^1^*, Tianxi Liu^1,6^ and Klaus Müllen^3,7^

^1^State Key Laboratory of Advanced Fiber Materials, College of Materials Science and Engineering, Donghua University, 2999 North Renmin Road, Shanghai 201620, P. R. China

^2^State Key Laboratory of Advanced Fiber Materials, College of Chemistry and Chemical Engineering, Donghua University, 2999 North Renmin Road, Shanghai 201620, P. R. China

^3^Nanotechnology Centre, Centre for Energy and Environmental Technologies, VŠB–Technical University of Ostrava, 17. listopadu 2172/15, Ostrava-Poruba 70800, Czech Republic

^4^Department of Chemistry, KU Leuven, Celestijnenlaan 200F, Leuven 3001, Belgium

^5^State Key Laboratory of Metal Matrix Composites, School of Materials Science and Engineering, Shanghai Jiao Tong University, Shanghai 200240, P. R. China

^6^Key Laboratory of Synthetic and Biological Colloids, Ministry of Education, School of Chemical and Material Engineering, Jiangnan University, Wuxi, 214122, P. R. China

^7^Department of Molecular Spectroscopy, Max Planck Institute for Polymer Research, Ackermannweg 10, Mainz 55128, Germany

*Corresponding authors. E-mail: [zhanghui@dhu.edu.cn](mailto:zhanghui@dhu.edu.cn) (Hui Zhang); [feililai@sjtu.edu.cn](mailto:feililai@sjtu.edu.cn) (Feili Lai); [yuee_miao@dhu.edu.cn](mailto:yuee_miao@dhu.edu.cn) (Yue-E Miao)

**S1 Supplementary Figures and Tables**


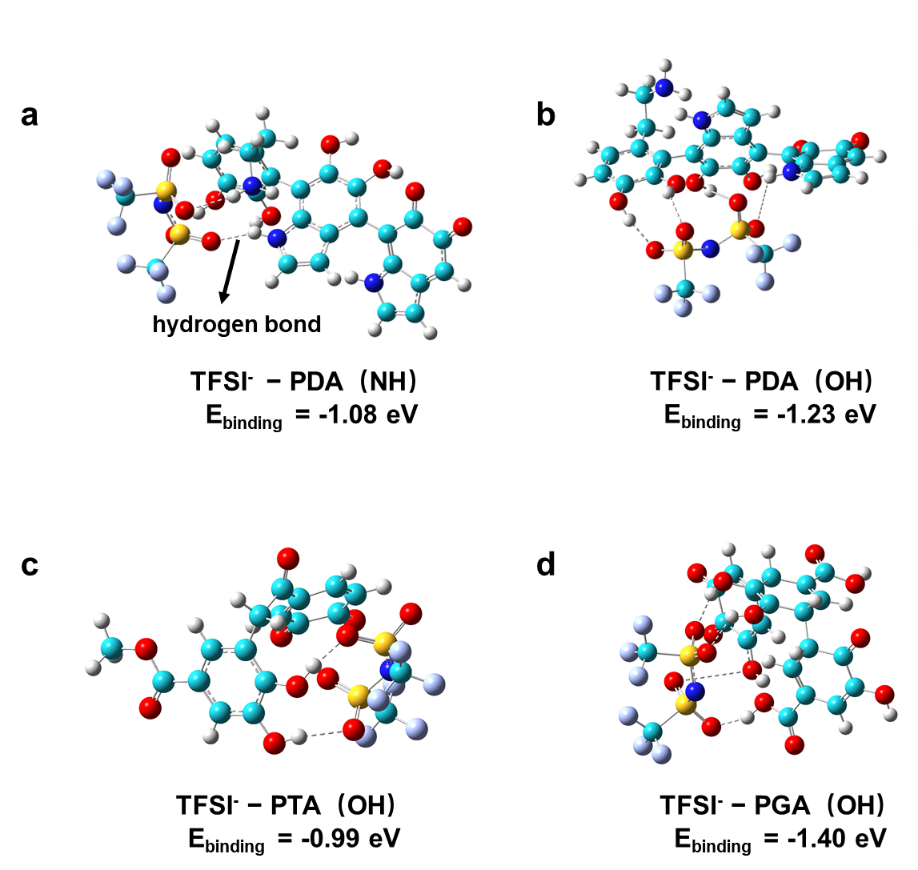


**Fig. S1** DFT-calculated binding energies between TFSI⁻ anion and the polar groups in **a, b** PDA, **c** PTA and **d** PGA, respectively


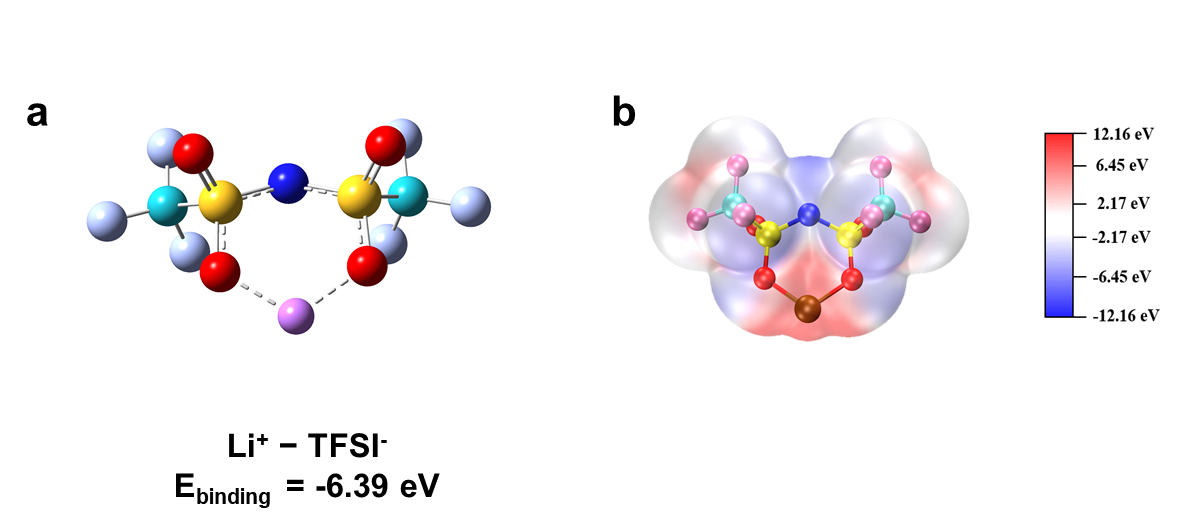


**Fig. S2 a** Optimized geometrical configurations and binding energies between Li^+^ and TFSI^−^ calculated by DFT. **b** ESP map of the Li^+^-TFSI⁻ solvation structure


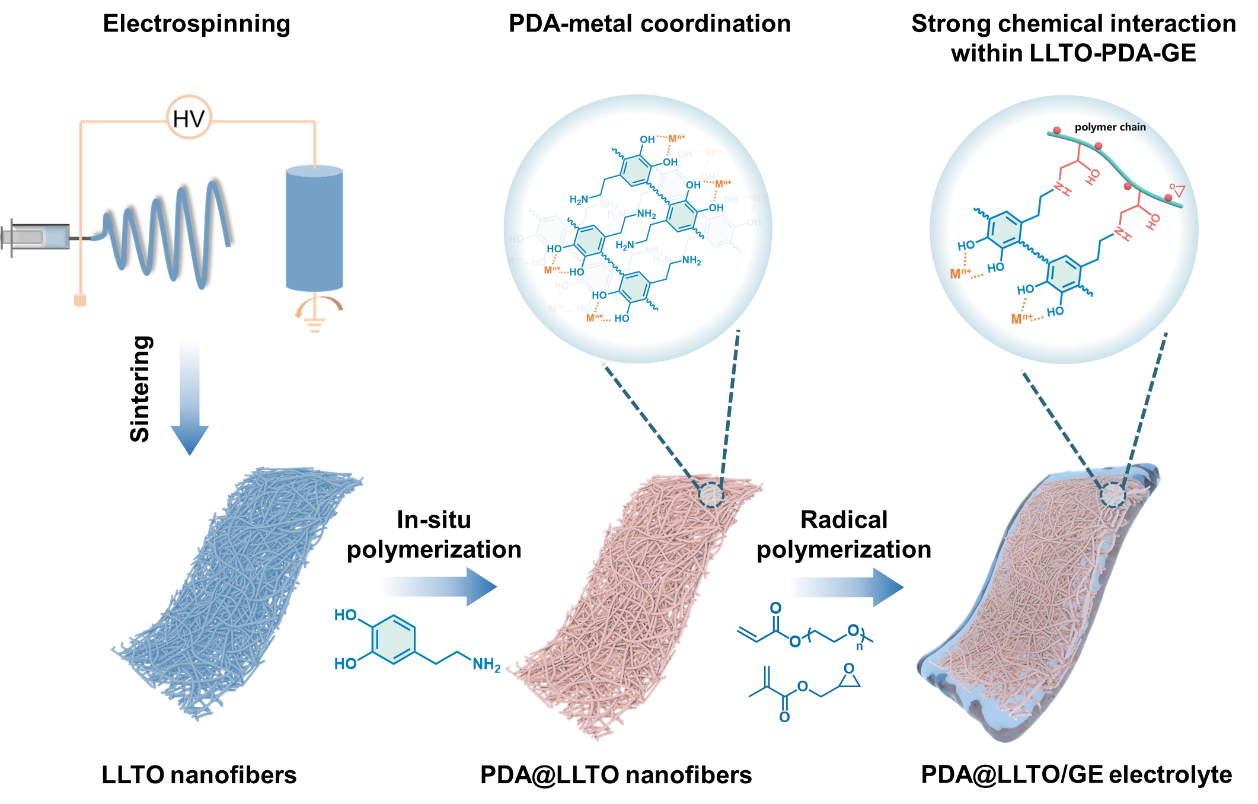


**Fig. S3** Schematic illustration of the preparation process for the PDA@LLTO/GE electrolyte


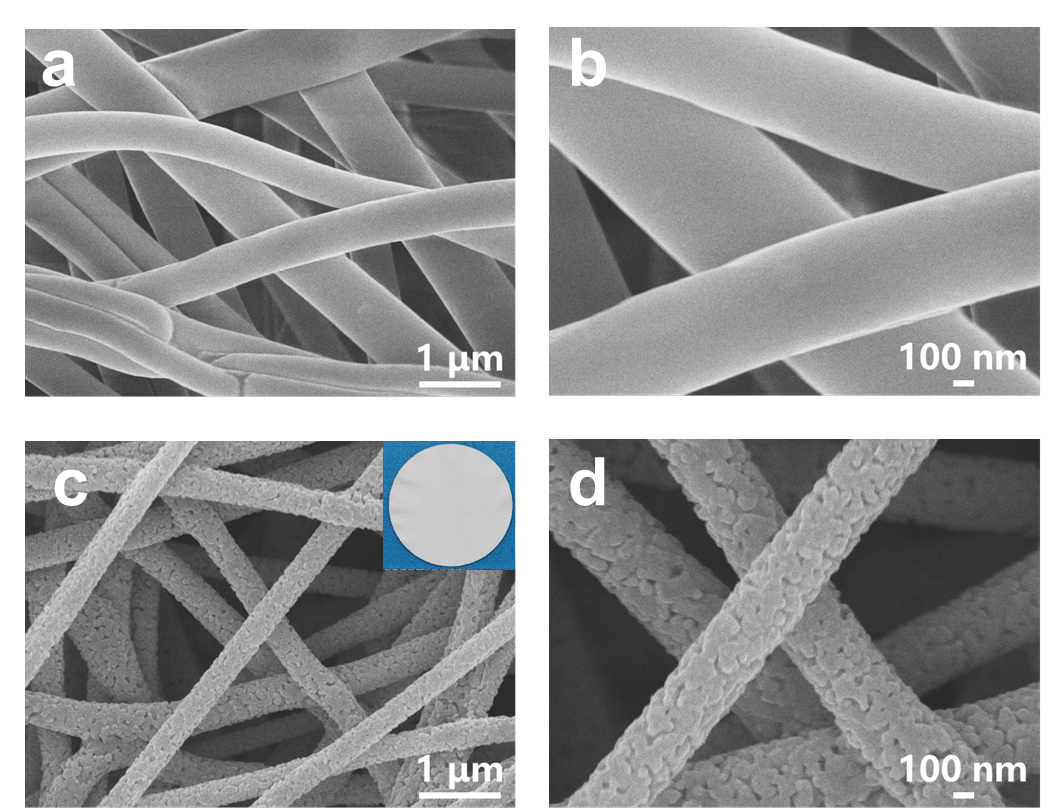


**Fig. S4** SEM images of a, b p-LLTO nanofibers, and **c, d** LLTO nanofibers. The inset in **c** shows a digital photograph of the LLTO membrane


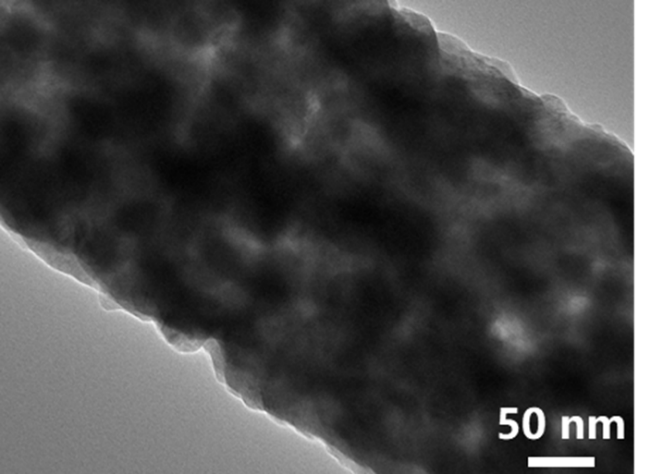


**Fig. S5** TEM image of LLTO nanofiber


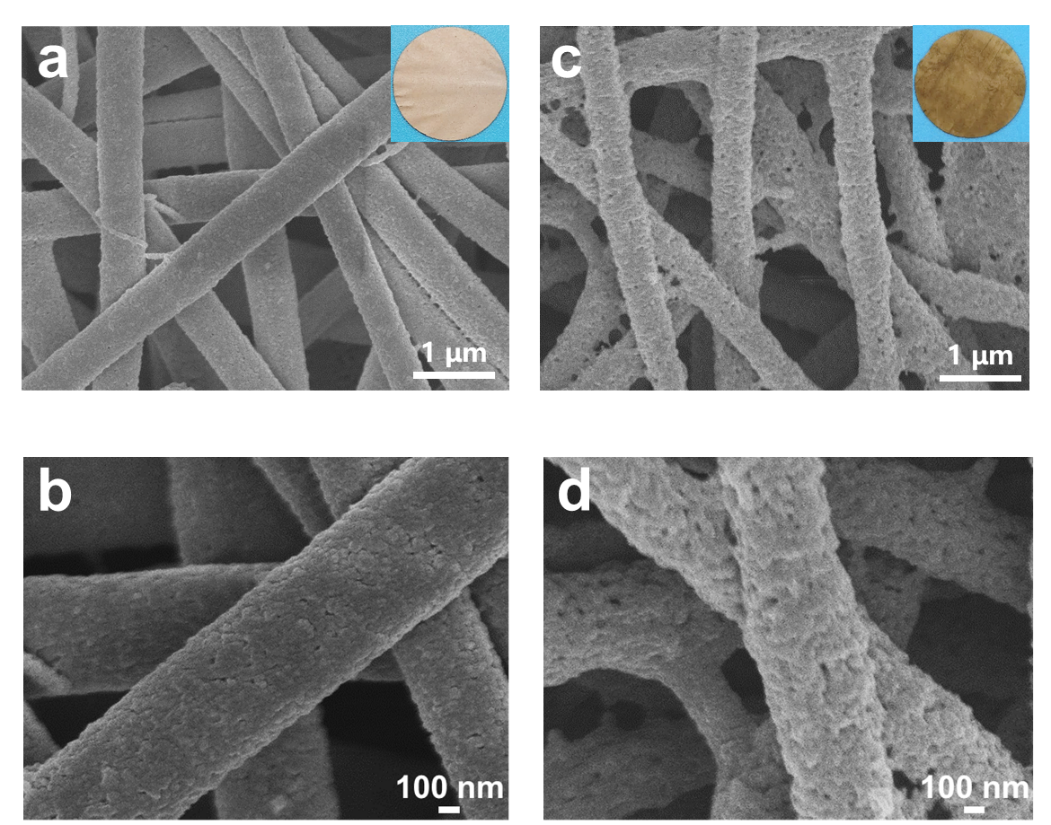


**Fig. S6** SEM images of PDA_x_@LLTO nanofibers with different DA concentrations of **a, b** 0.5 mg mL⁻^1^ and **c, d** 4 mg mL⁻^1^. The insets in **a** and **c** show the corresponding digital photographs


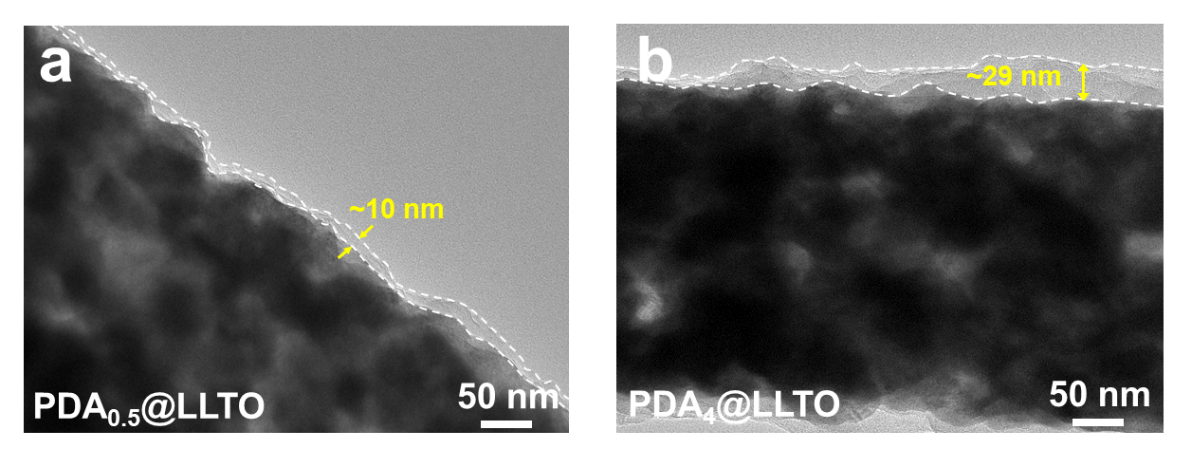


**Fig. S7** TEM images of **a** PDA_0.5_@LLTO, and **b** PDA_4_@LLTO nanofibers


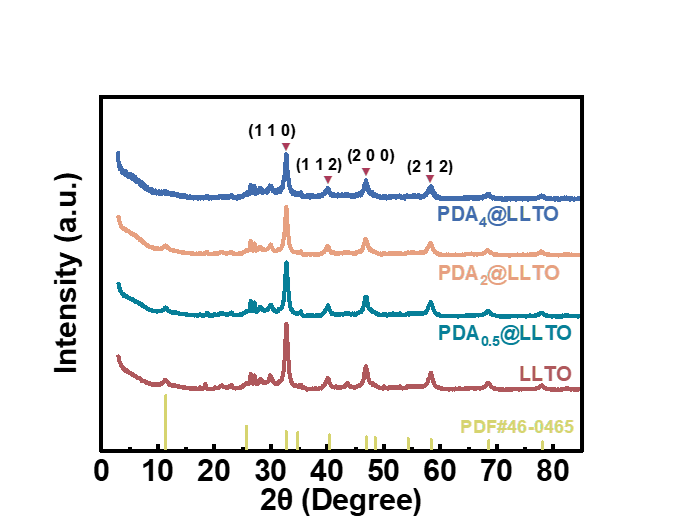


**Fig. S8** XRD patterns of LLTO and PDA_x_@LLTO membranes

**Fig. S9** High-resolution La 3*d* XPS spectra of LLTO and PDA_2_@LLTO membranes

**Fig. S10** TGA curves of LLTO/GE and PDA_x_@LLTO/GE electrolytes

**Fig. S11** DSC curves of LLTO/GE and PDA_x_@LLTO/GE electrolytes


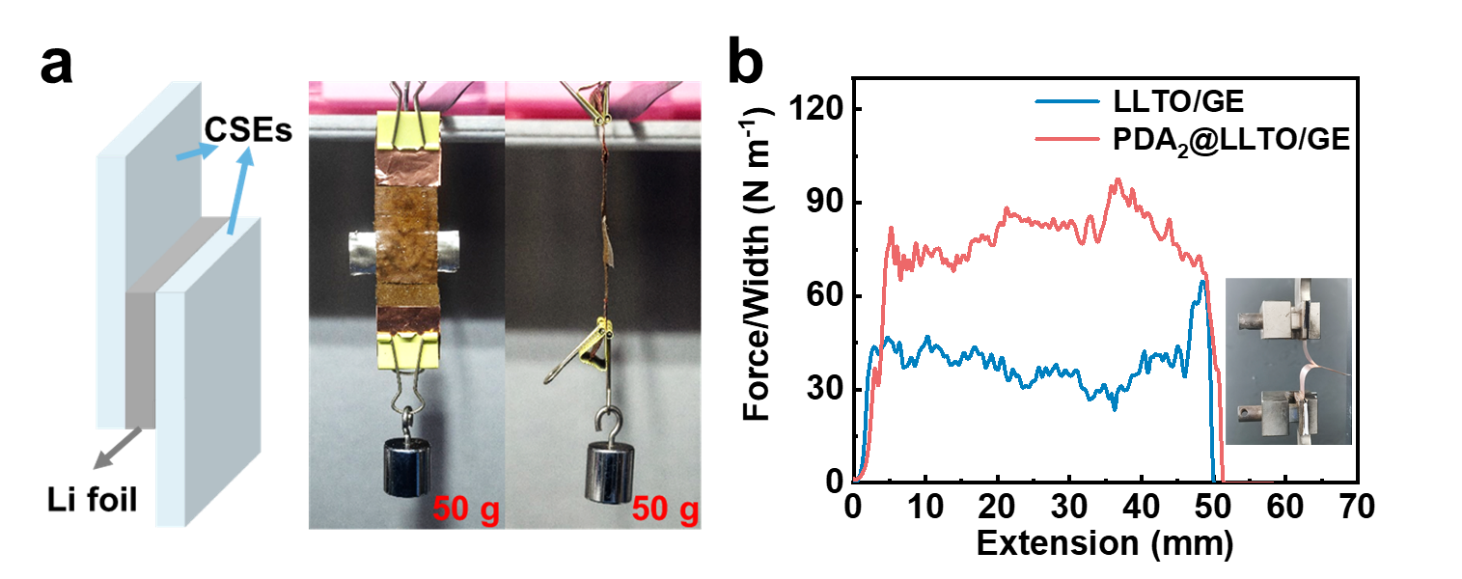


**Fig. S12 a** Digital photographs of the PDA_2_@LLTO/GE electrolyte holding a 50 g weight while adhered to a Li metal foil. **b** The interfacial adhesion comparison between LLTO/GE and PDA_2_@LLTO/GE electrolytes


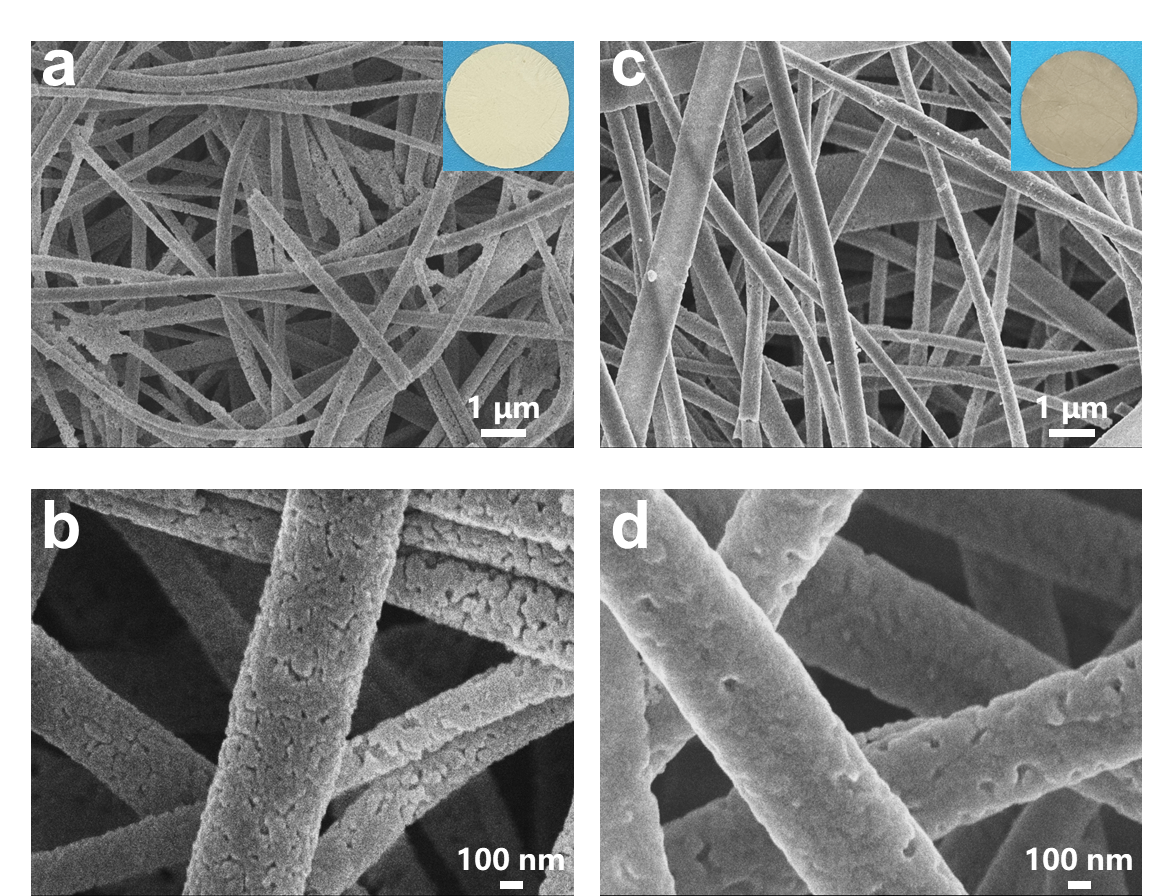


**Fig. S13** SEM images of **a, b** PTA_2_@LLTO and **c, d** PGA_2_@LLTO nanofibers. The insets in **a** and **c** show the corresponding digital images


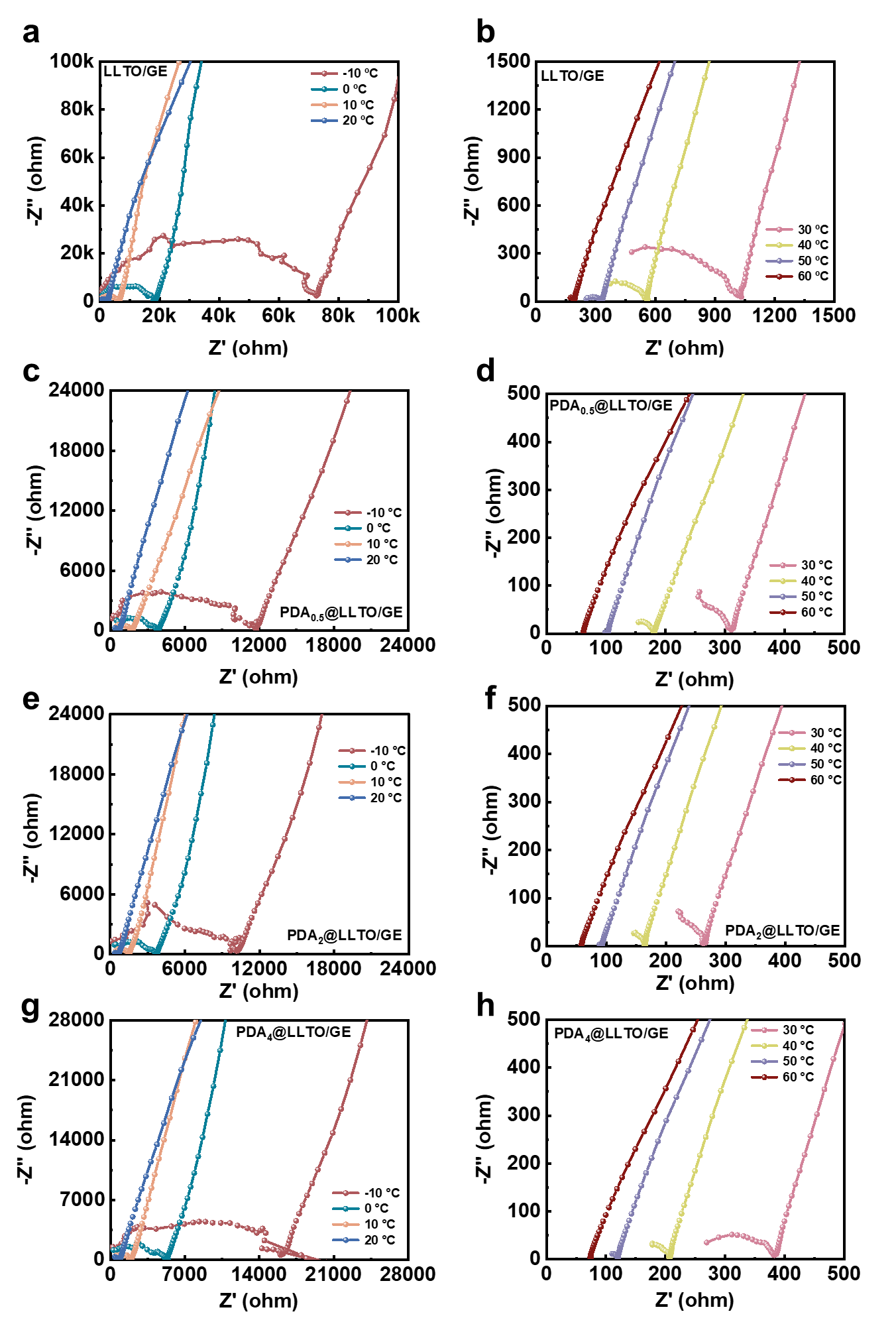


**Fig. S14** EIS spectra of **a, b** LLTO/GE, and **c-h** PDA_x_@LLTO/GE electrolytes at different temperature ranging from –10 to 60 ℃


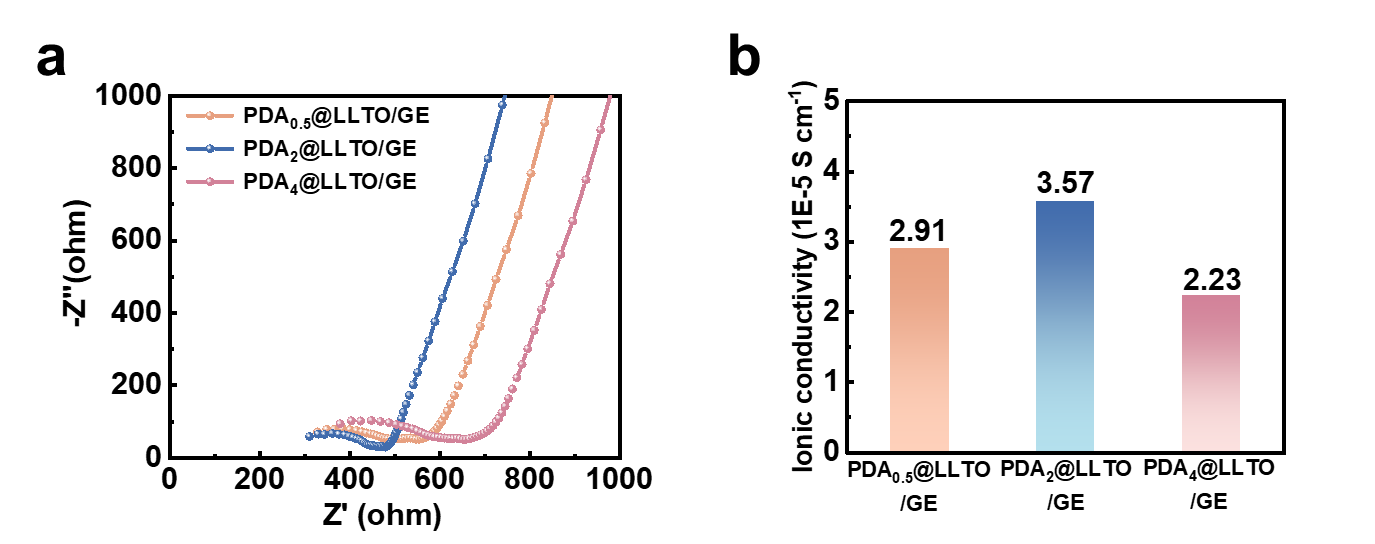


**Fig. S15** **a** EIS spectra of PDA_x_@LLTO/GE electrolytes at room temperature. **b** The corresponding ionic conductivities of the electrolytes


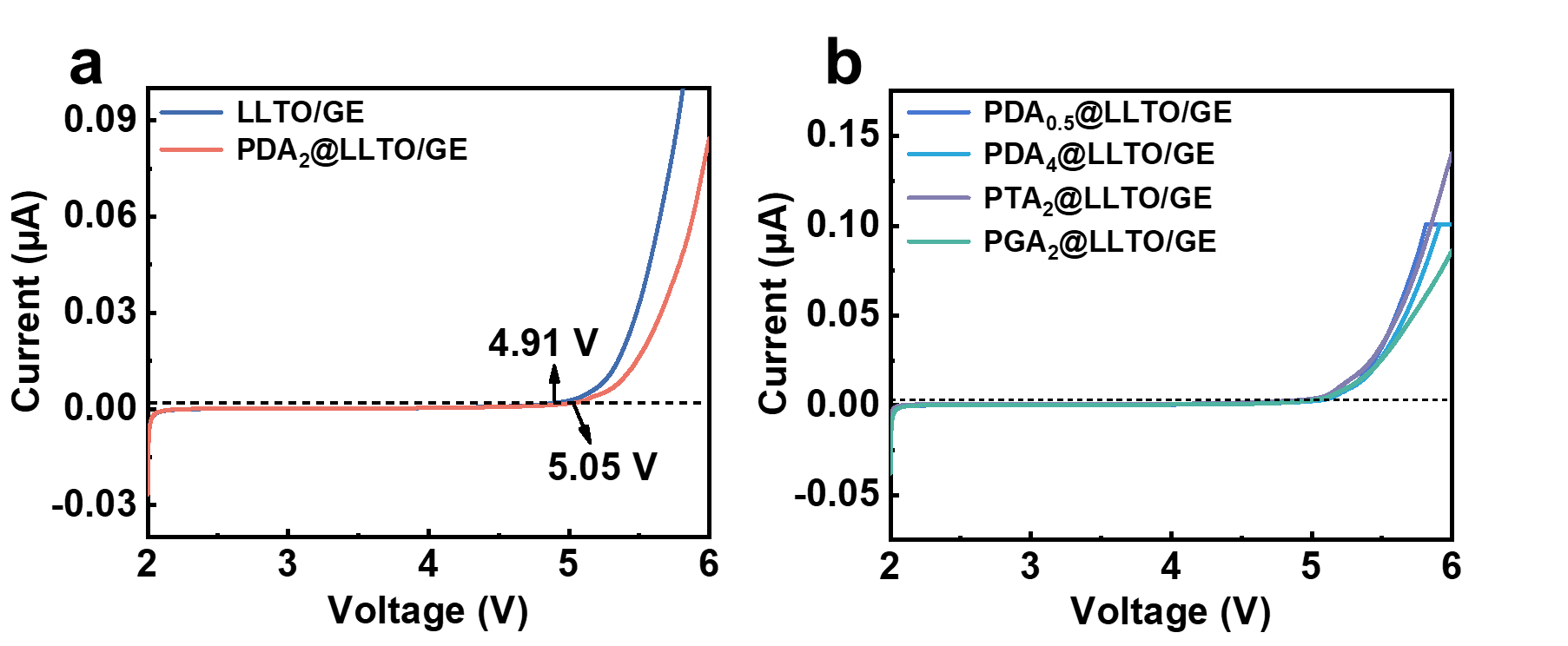


**Fig. S16** LSV curves of different electrolytes


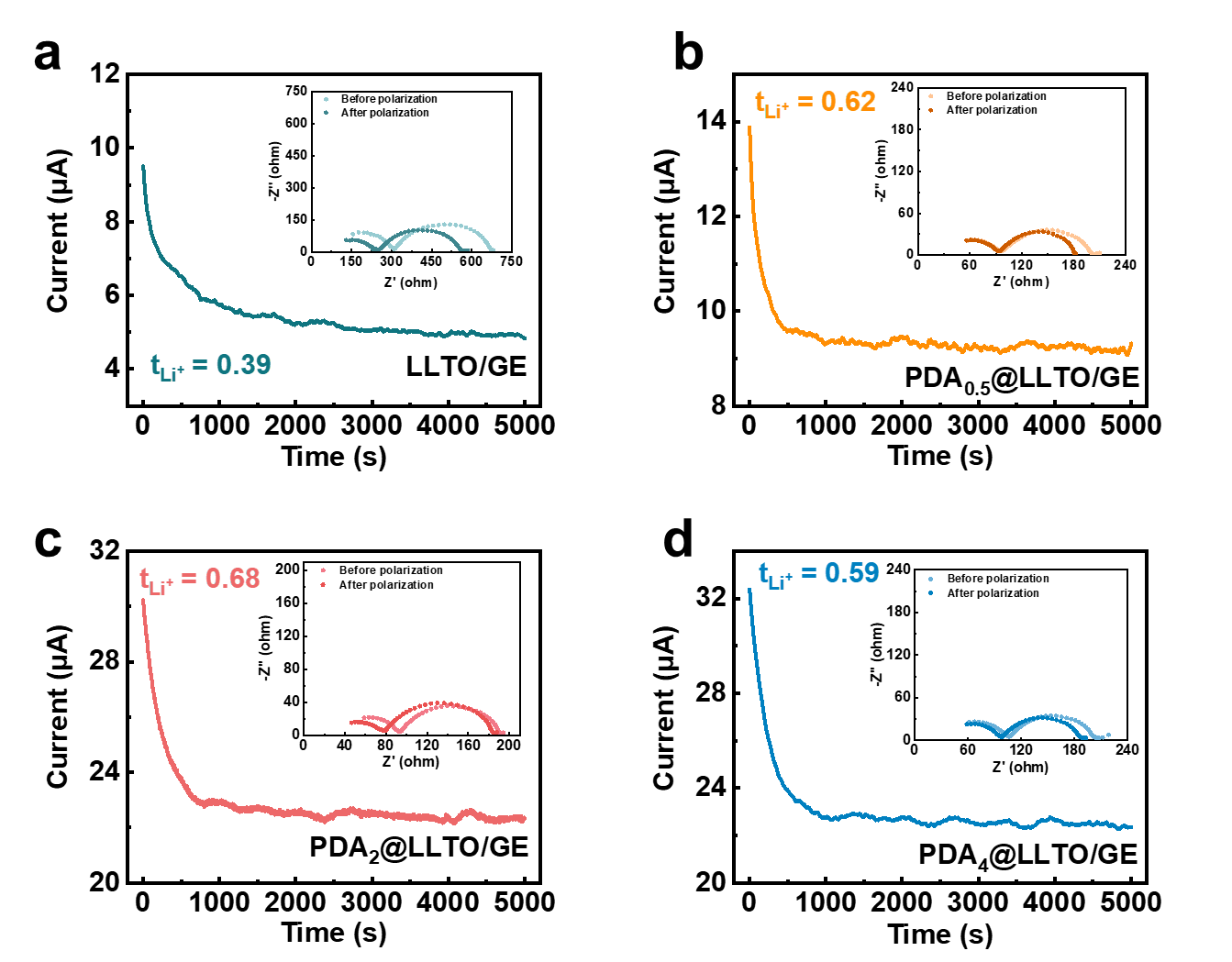


**Fig. S17** Chronoamperometry curves of cells at 60 ℃ using **a** LLTO/GE, and **b-d** PDA_x_@LLTO/GE electrolytes under a step voltage of 10 mV. Insets display the corresponding EIS plots before and after polarization


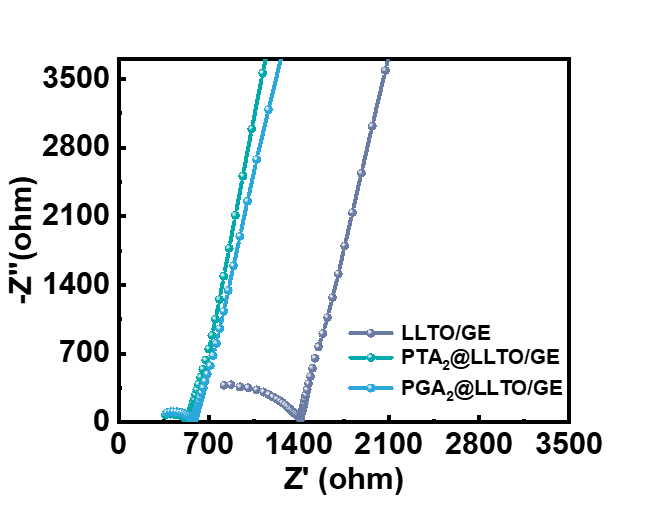


**Fig. S18** EIS plots of different electrolytes at room temperature


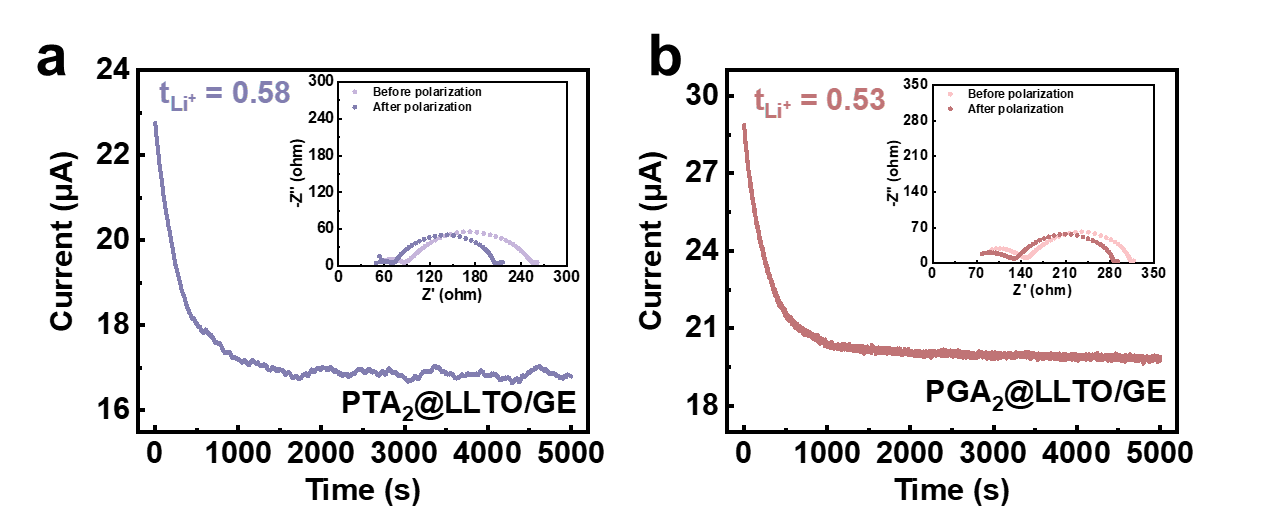


**Fig. S19** Chronoamperometry curves of cells at 60 ℃ using **a** PTA_2_@LLTO/GE, and **b** PGA_2_@LLTO/GE under a step voltage of 10 mV. Insets display the corresponding EIS plots before and after polarization


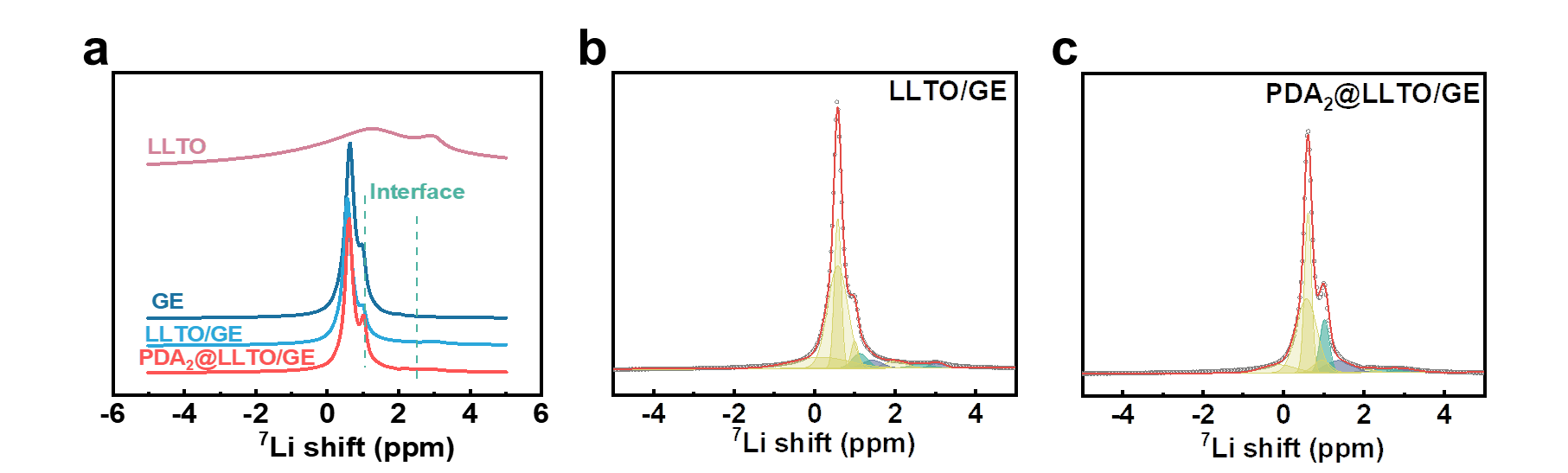


**Fig. S20 a** Solid-state ^7^Li NMR spectra of LLTO, GE, LLTO/GE and PDA_2_@LLTO/GE electrolytes. Partially enlarged diagrams from the solid-state ^7^Li NMR spectra of **b** LLTO/GE, and **c** PDA_2_@LLTO/GE electrolytes


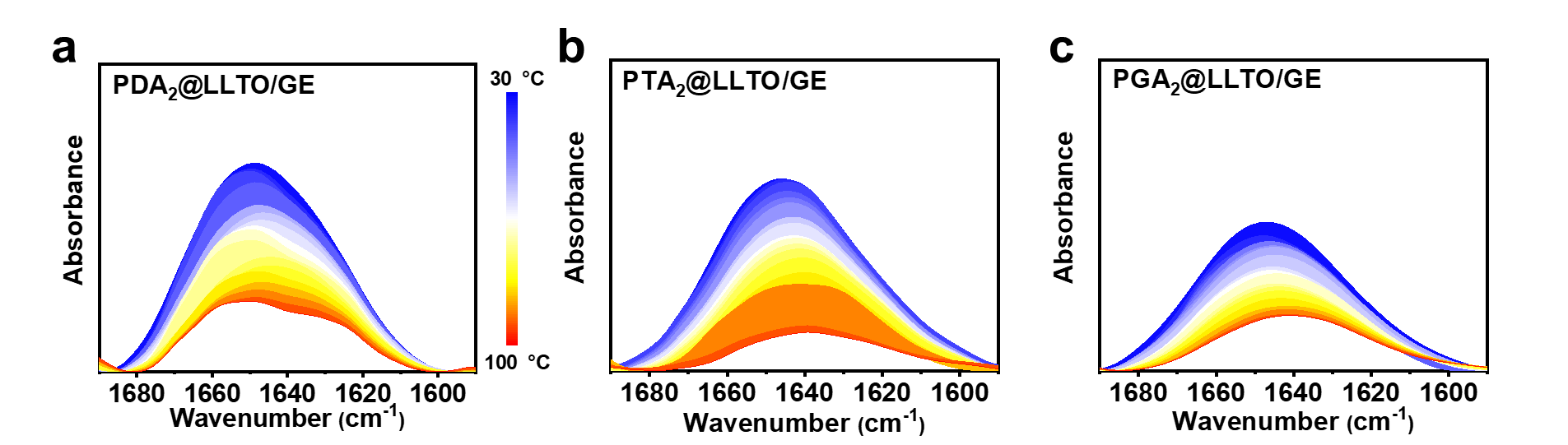


**Fig S21** Temperature-variable FTIR spectra of **a** PDA_2_@LLTO/GE, **b** PTA_2_@LLTO/GE and **c** PGA_2_@LLTO/GE recorded in the range of 1690 - 1590 cm^−1^ during heating from 30 to 100 °C


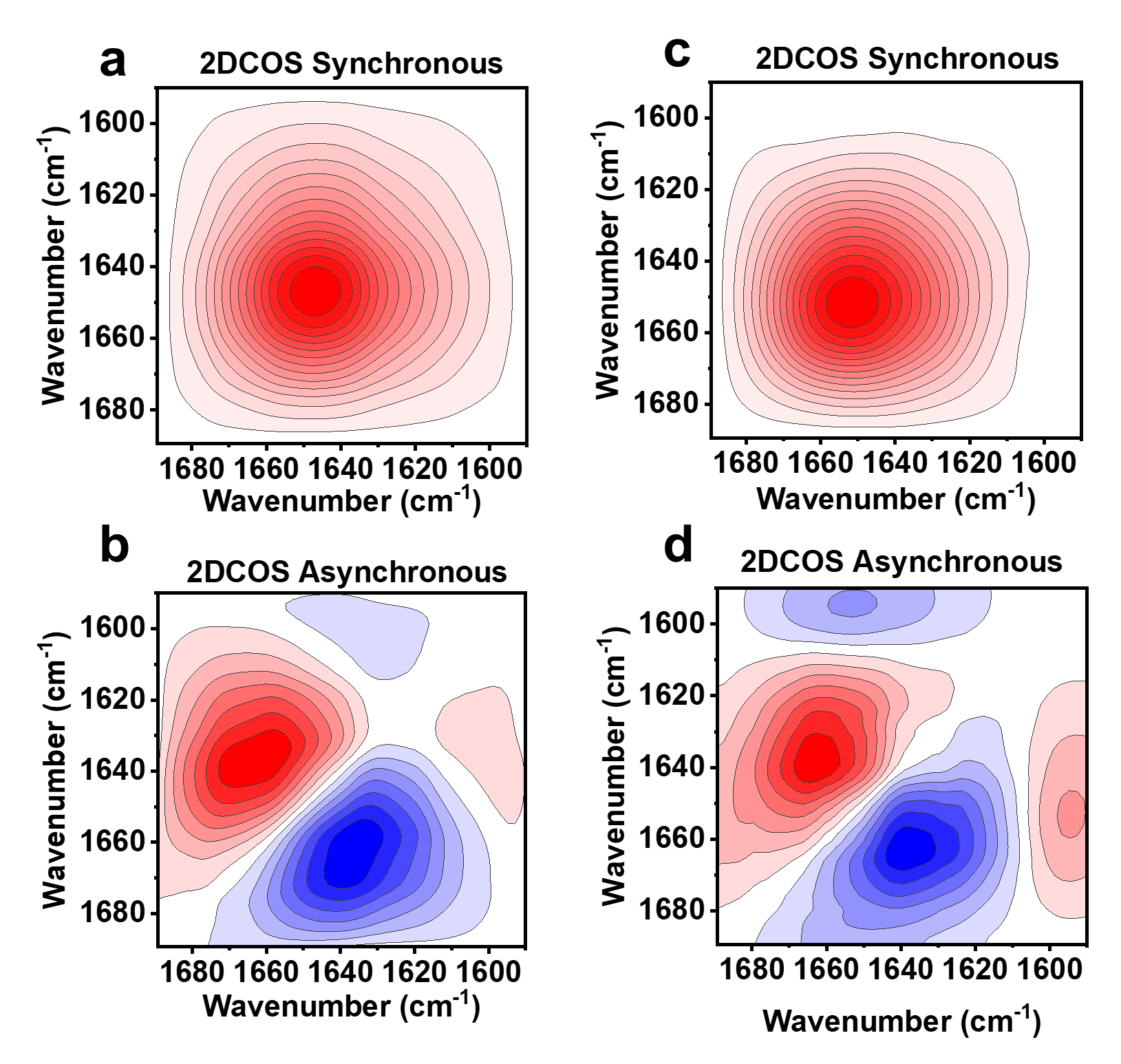


**Fig. S22** 2DCOS **a, c** synchronous and **b, d** asynchronous spectra of PTA_2_@LLTO/GE and PGA_2_@LLTO/GE electrolyte, respectively. Warm colors (red) represent positive intensities, while cold colors (blue) indicate negative intensities


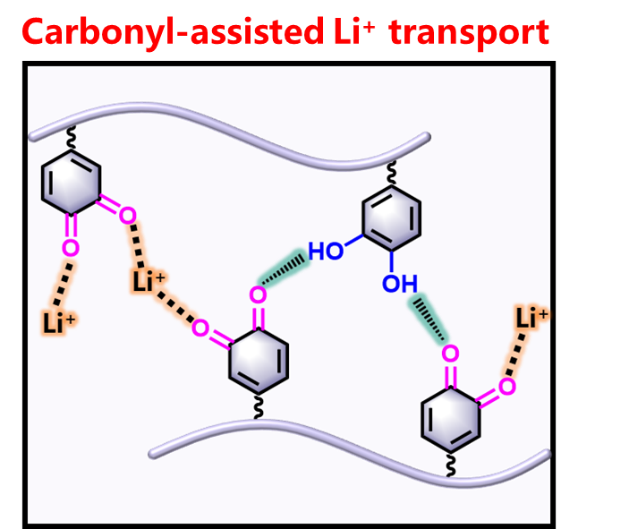


**Fig. S23** Schematic diagrams of the carbonyl-assisted Li^+^ transport modes at the polyphenol interfaces


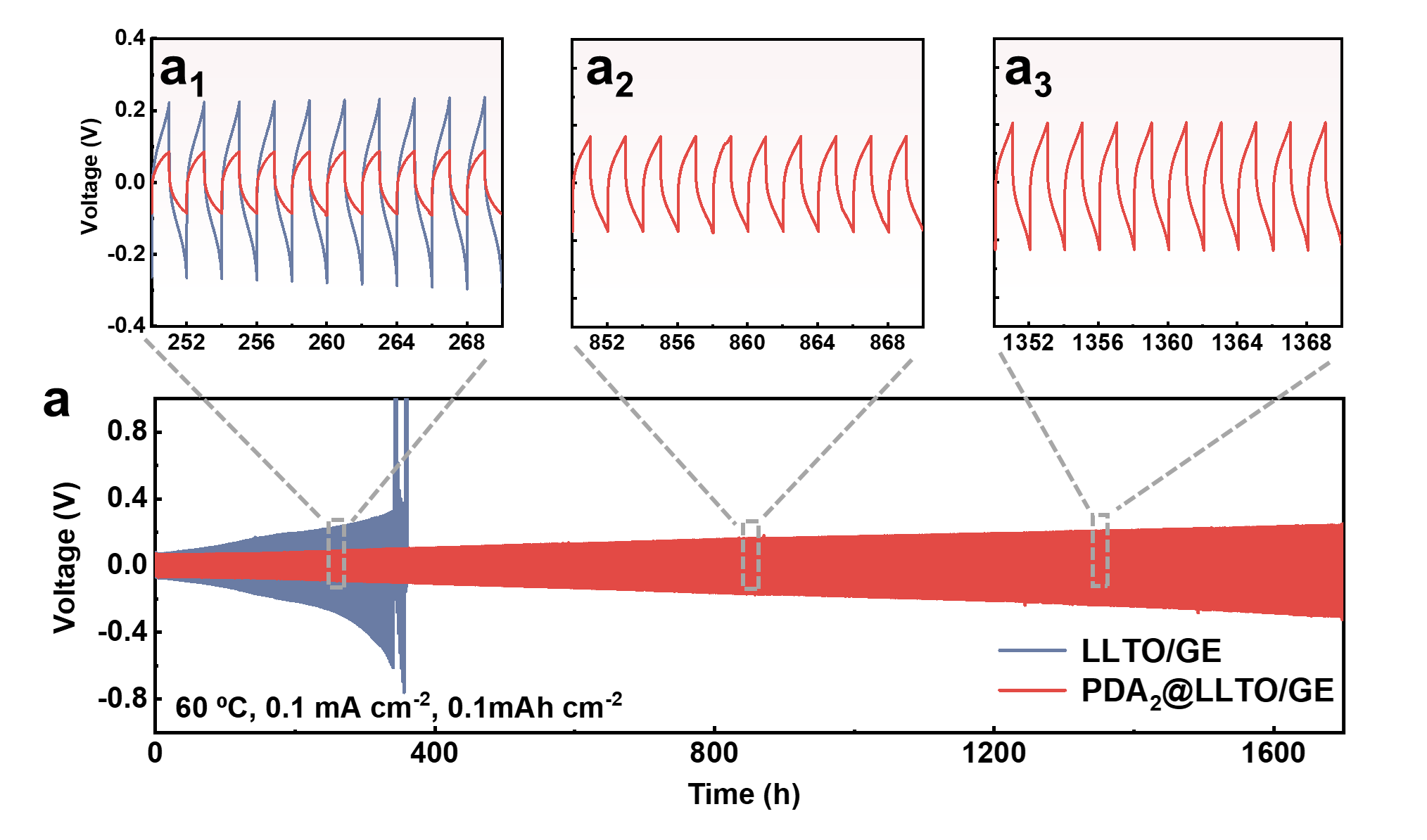


**Fig. S24** Galvanostatic curves of symmetric cells assembled with LLTO/GE and PDA_2_@LLTO/GE electrolytes at 0.1 mA cm⁻^2^ and 60 ℃, (a_1_–a_3_) show enlarged view of the voltage–time curves during the periods of 250-270, 850-870 and 1350-1370 h, respectively


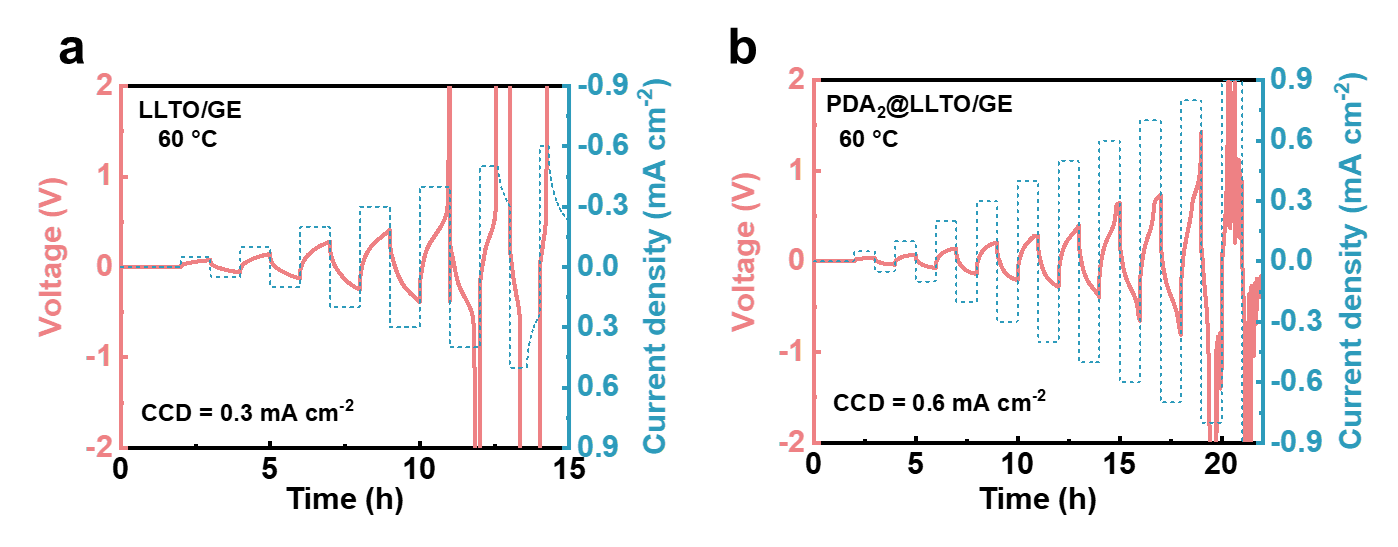


**Fig. S25** CCD tests of **a** LLTO/GE and **b** PDA_2_@LLTO/GE, respectively

**
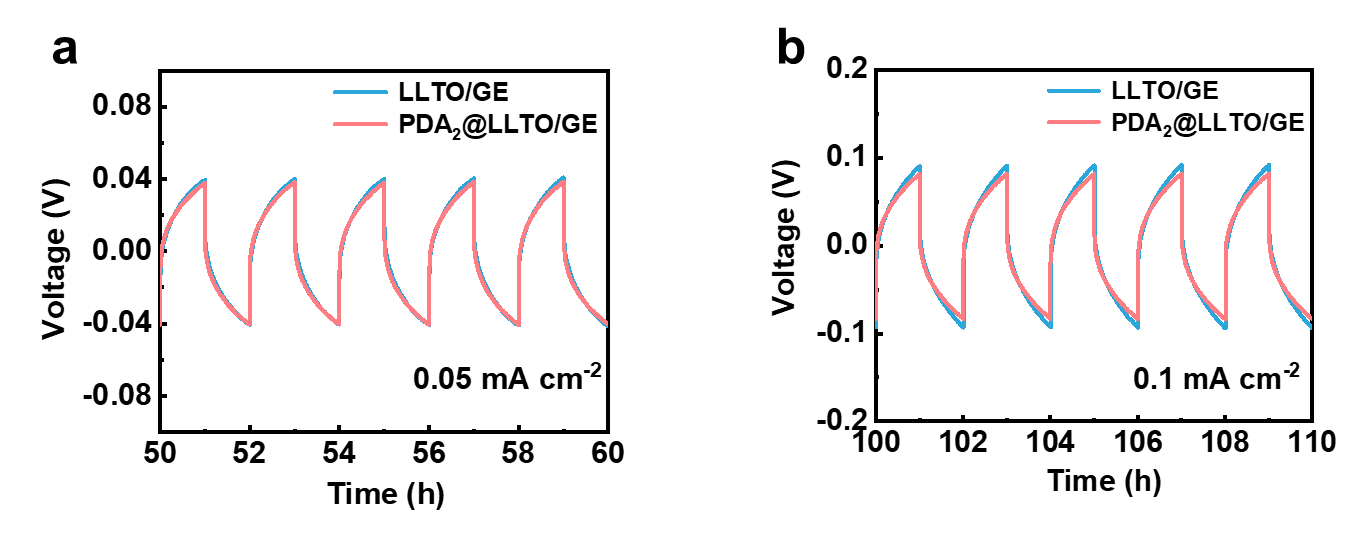
**

**Fig. S26** The enlarged views of the voltage-time curves of LLTO/GE and PDA_2_@LLTO/GE at different current density of **a** 0.05 mA cm⁻^2^ and **b** 0.1 mA cm⁻^2^, respectively


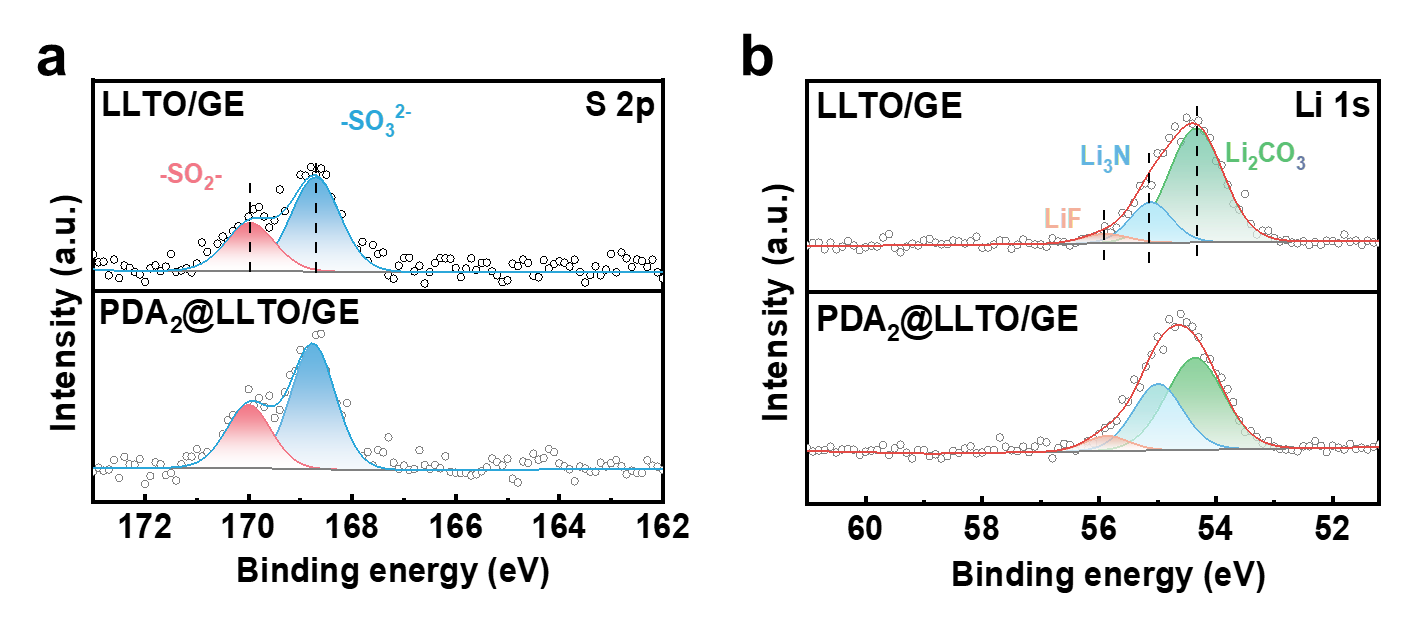


**Fig. S27** XPS spectra of the lithium surface after 20 cycles with LLTO/GE and PDA_2_@LLTO/GE electrolytes: **a** S 2*p*, **b** Li 1*s*


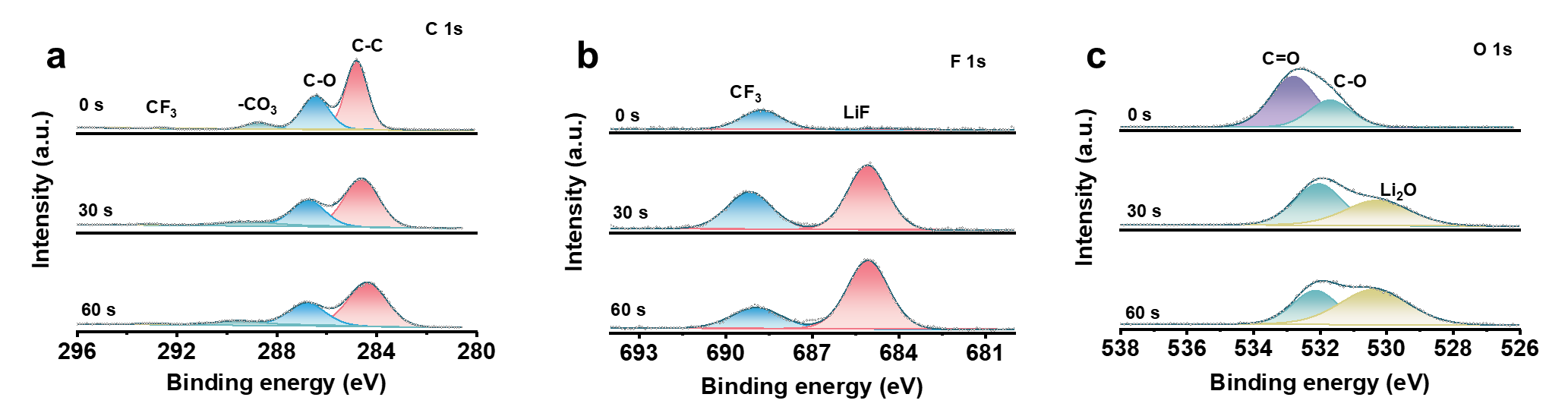


**Fig. S28** Depth-profiling XPS spectra of **a** C 1*s*, **b** F 1*s* and **c** O 1*s* at different etching depths for the SEI layer on Li anodes disassembled from Li || Li cell assembled with PDA_2_@LLTO/GE after 100 cycles at 0.2 mA cm⁻^2^


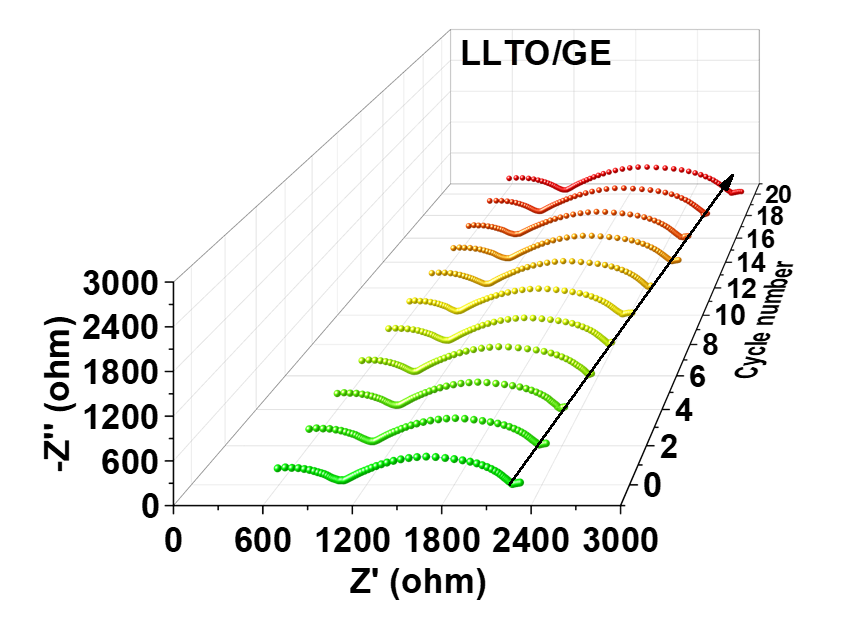


**Fig. S29** EIS plots of Li | LLTO/GE | Li at different cycles


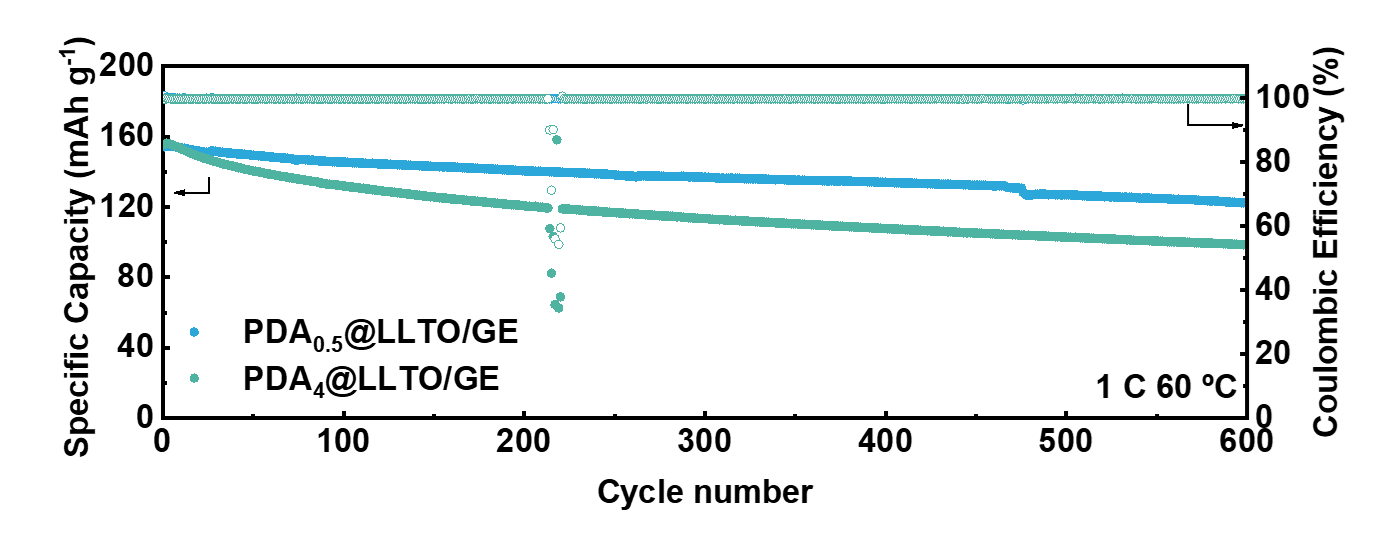


**Fig. S30** Long-term cycling properties of LFP| PDA_0.5_@LLTO/GE |Li and LFP| PDA_4_@LLTO/GE |Li full batteries under 1 C at 60 ℃


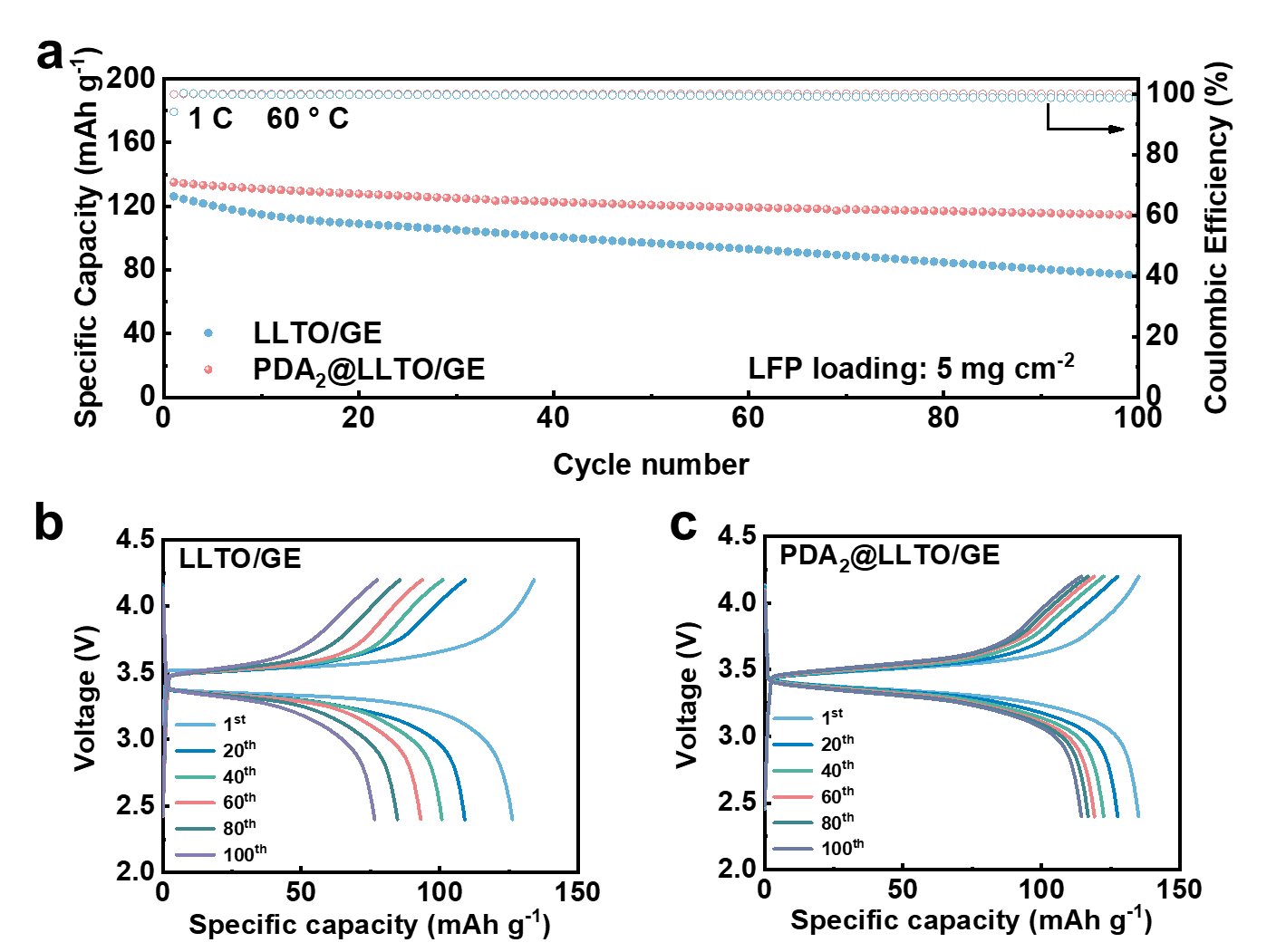


**Fig. S31** **a** Cycling performance of LFP| LLTO/GE |Li and LFP| PDA_2_@LLTO/GE |Li full batteries with a high LFP loading of 5 mg cm⁻^2^ under 1 C at 60 ℃. The corresponding charge-discharge curves of **b** LLTO/GE and **c** PDA_2_@LLTO/GE

**Fig. S32** Charge/discharge profiles of the LFP| LLTO/GE |Li cell under the varying current density from 0.5 to 5 C at 60 ℃

**
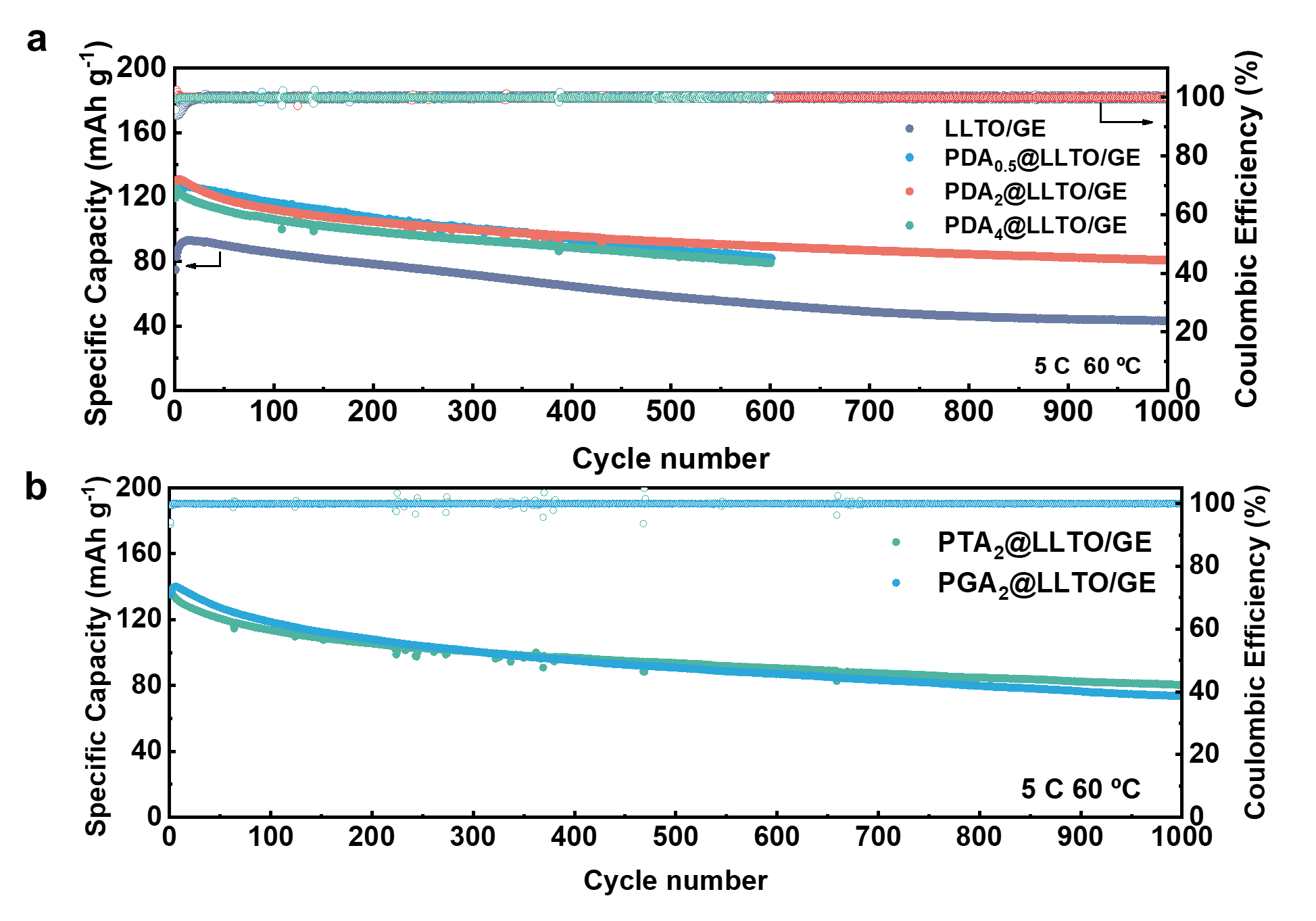
**

**Figure S33.** Long-term cycling properties of full batteries of **a** LFP| LLTO/GE |Li and LFP| PDA_x_@LLTO/GE |Li and **b** LFP| PTA_2_@LT/GE |Li and LFP| PGA_2_@LLTO/GE |Li under 5 C at 60 ℃


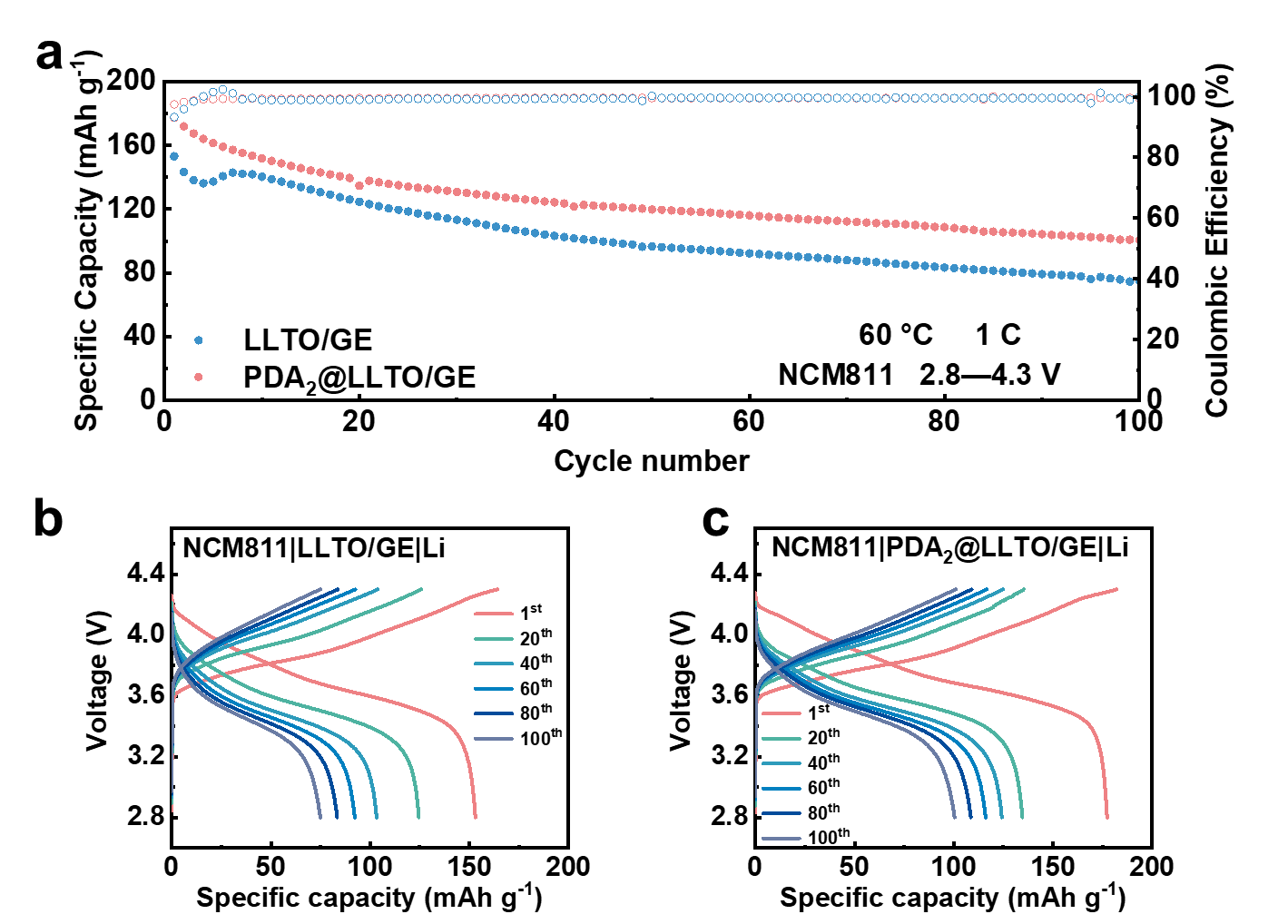


**Fig. S34** **a** Cycling performance of the high voltage NCM811 full batteries assembled with LLTO/GE and PDA_2_@LLTO/GE at 1 C at 60 ℃. The corresponding charge–discharge profiles for **b** LLTO/GE and **c** PDA_2_@LLTO/GE, respectively


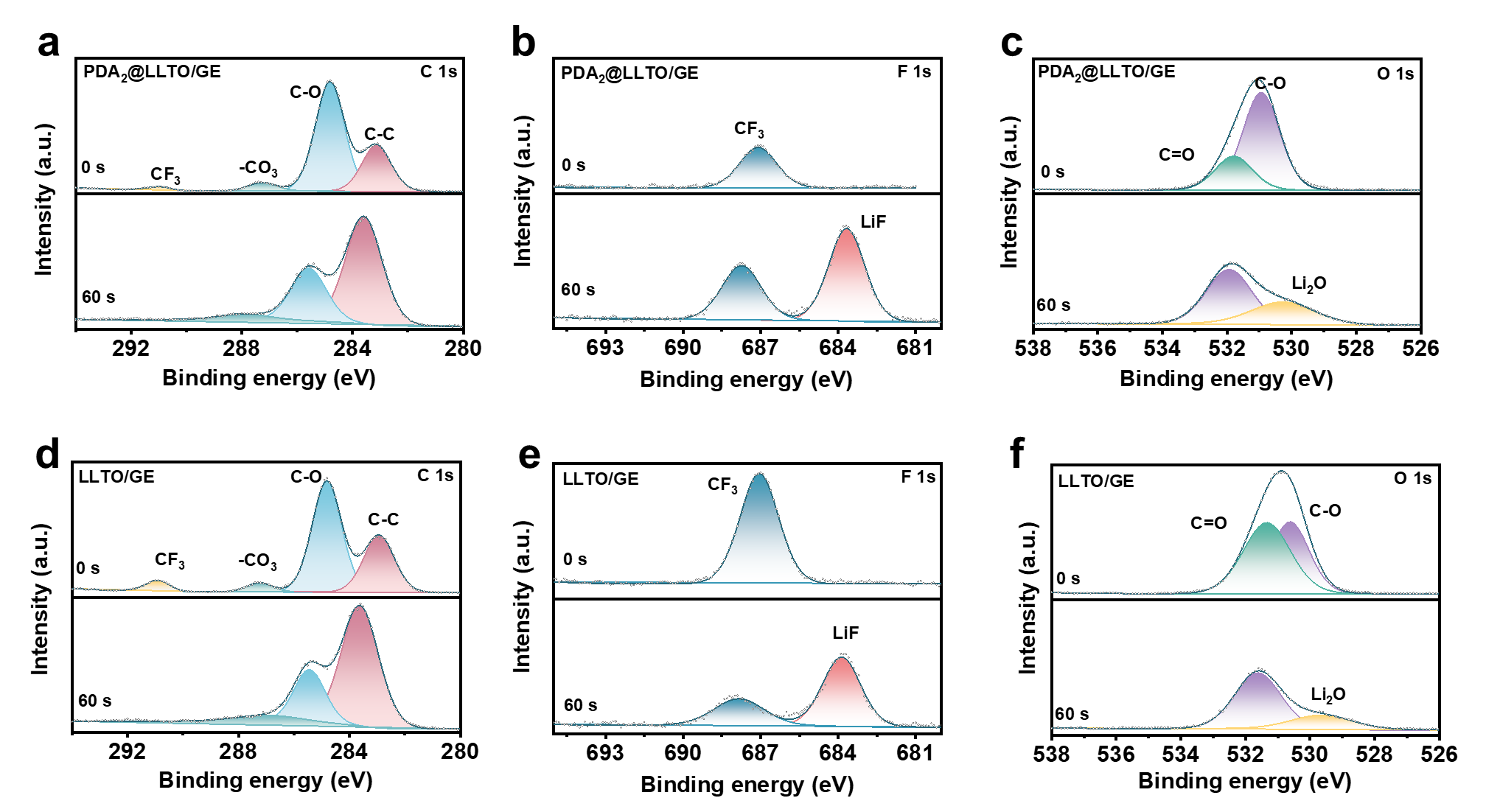


**Fig. S35** XPS spectra of the CEI layers in the NCM811| PDA_2_@LLTO/GE |Li and NCM811| LLTO/GE |Li cells: **a, d** C 1*s*, **b, e** F 1*s*, and **c, f** O 1*s*

**Table S1** Signs of the main cross-peaks in 2DCOS synchronous and asynchronous spectra. Signs read in synchronous and asynchronous spectra (left: Syn; right: Asyn)

| PDA_2_@LLTO/GE | | | |
| --- | --- | --- | --- |
| 1629 | +,+ | +,+ | C=O…Li^+^ |
| 1648 | +,+ | C=O…H-X |  |
| 1668 | C=O…C=O |  |  |
|  | 1668 | 1648 | 1629 |
| PGA_2_@LLTO/GE | | | |
| 1627 | +,+ | +,+ | C=O…Li^+^ |
| 1647 | +,+ | C=O…H-X |  |
| 1664 | C=O…C=O |  |  |
|  | 1664 | 1647 | 1627 |
| PTA_2_@LLTO/GE | | | |
| 1621 | +,+ | +,+ | C=O…Li^+^ |
| 1646 | +,+ | C=O…H-X |  |
| 1662 | C=O…C=O |  |  |
|  | 1662 | 1646 | 1621 |
| C=O…Li^+^ > C=O…H-X > C=O…C=O | | | |

Based on Noda’s rule, the responsive order of different wavenumbers is determined to be: 1668 → 1648 → 1629 cm^-1^, 1664 → 1647 → 1627 cm^-1^, and 1662 → 1646 → 1621 cm^-1^ for D@LT/GE, G@LT/GE and T@LT/GE electrolytes, respectively.
